# TIPP3 and TIPP3-fast: Improved abundance profiling in metagenomics

**DOI:** 10.1371/journal.pcbi.1012593

**Published:** 2025-04-04

**Authors:** Chengze Shen, Eleanor Wedell, Mihai Pop, Tandy Warnow

**Affiliations:** 1 Siebel School of Computing and Data Science, University of Illinois Urbana-Champaign, Urbana, Illinois, United States of America; 2 Department of Computer Science, University of Maryland at College Park, College Park, Maryland, United States of America; Fudan University, CHINA

## Abstract

We present TIPP3 and TIPP3-fast, new tools for abundance profiling in metagenomic datasets. Like its predecessor, TIPP2, the TIPP3 pipeline uses a maximum likelihood approach to place reads into labeled taxonomies using marker genes, but it achieves superior accuracy to TIPP2 by enabling the use of much larger taxonomies through improved algorithmic techniques. We show that TIPP3 is generally more accurate than leading methods for abundance profiling in two important contexts: when reads come from genomes not already in a public database (i.e., novel genomes) and when reads contain sequencing errors. We also show that TIPP3-fast has slightly lower accuracy than TIPP3, but is also generally more accurate than other leading methods and uses a small fraction of TIPP3’s runtime. Additionally, we highlight the potential benefits of restricting abundance profiling methods to those reads that map to marker genes (i.e., using a filtered marker-gene based analysis), which we show typically improves accuracy. TIPP3 is freely available at https://github.com/c5shen/TIPP3.

## Author summary

TIPP3 is a new method for abundance profiling based on marker genes (i.e., genes that are universal and single copy) that improves on its predecessors, TIPP and TIPP2. By using advances in multiple sequence alignment and phylogenetic placement, TIPP3 is able to work with substantially larger reference packages (i.e., multiple sequence alignments and taxonomies for each of its marker genes) than in TIPP2, so that these contain  ∼ 55,000 sequences per marker gene. This extension to large reference packages produces improved accuracy in abundance profiling compared to TIPP2 and also provides advantages over the current leading abundance profiling methods. Specifically, TIPP3 achieves higher accuracy than competing methods when input reads have high sequencing errors or are from novel genomes. The fast version, TIPP3-fast, is slightly less accurate than TIPP3 but nevertheless useful: compared to the most accurate competing methods, it is competitive in runtime and more accurate under challenging conditions. Finally, we show improvements to Kraken and Bracken when the input reads are filtered to those drawn from the marker genes. TIPP3 is open-source and available at https://github.com/c5shen/TIPP3.

## Introduction

Understanding the complex interactions between microorganisms in their communities has become critical to human and environmental health studies [1–4]. Typically, the first step in understanding these interactions (i.e., microbiome analysis) is taxonomic profiling, which identifies and quantifies the relative abundance of species in a microbial community.

Some studies estimate abundance profiles using amplified 16S ribosomal RNA present in prokaryotic species [5], which is cost-effective but can have quantification errors introduced by copy number variation [6]. As sequencing costs decrease continuously, newer methods for taxonomic profiling increasingly use metagenomic data, which consists of DNA reads sequenced directly from the target microbial environment, capturing millions of sequences from all genomes in the community.

Current methods for taxonomic profiling vary in their approach to using metagenomic data. Methods such as Kraken [7,8], Bracken [9], and more recently Metabuli [10] are k-mer based and map input reads for classification to their databases, which consist of sequenced microbial genomes. Other methods, including MetaPhyler [11], MetaPhlAn [12–14], mOTUs [15], TIPP [16], and TIPP2 [17], use marker genes to assign reads to microbial clades for classification and abundance profiling, since marker genes are single-copy and universal in Bacteria and Archaea species. The reads that are classified by these methods are only those that have been assigned to a particular marker gene. Thus, the resulting estimated abundance does not need to be adjusted for genome size or copy number variation. Many methods use a reference database for read identification, but they may fail to identify reads of under-represented species. Some methods, such as TIPP [16] and TIPP2 [17], use maximum likelihood phylogenetic placement methods to place reads into reference taxonomic trees of marker genes, and use the location of the read in the taxonomy for taxonomic classification. This approach has the potential to enable the detection of distant homologs to reference sequences, allowing characterizations of highly diverse metagenomic reads [18]. TIPP and TIPP2 both use pplacer [19] for phylogenetic placement, which is a maximum likelihood phylogenetic placement method that has been shown to have very high accuracy [20,21]. TIPP2 differs from TIPP mainly by having denser taxon sampling for each marker gene, which results in improved accuracy. However, the way TIPP2 uses pplacer restricts its usage to trees with at most 10,000 sequences, and thus TIPP and TIPP2 are not scalable to large taxonomic trees [20–23].

In just the last few years, new phylogenetic placement methods have been developed that can place sequences into much larger reference trees [21–25]. In particular, SCAMPP [24] is a method that uses pplacer within a divide-and-conquer strategy so that it can scale to large trees, EPA-ng [18] is another maximum likelihood placement method that is close to the accuracy of pplacer but is much faster when there are many sequences to place, and BSCAMPP [25] is a method that uses EPA-ng within a divide-and-conquer strategy (not the same as used by SCAMPP) to enable it to scale to large trees. Given the improvement in accuracy obtained by TIPP2 over TIPP as a result of using a slightly larger taxonomic tree for each marker gene, we hypothesize that these improved phylogenetic placement methods could potentially lead to further improvements in abundance profiling accuracy.

In this study, we present TIPP3, an updated version of TIPP2. TIPP3 builds on TIPP2 and has a more extensively built reference package with 38 marker genes and more than 50,000 sequences per gene. TIPP3 also leverages the recent developments in more accurate multiple sequence alignment methods and scalable phylogenetic placement methods. We show empirically that TIPP3 is more accurate than TIPP2 for abundance profiling, particularly for lower taxonomic levels such as species, genus, and family. Compared to other leading profiling methods, TIPP3 is the most accurate under most conditions, especially for long reads with higher sequencing error (e.g., PacBio or Nanopore) and for reads from novel genomes. We also introduce TIPP3-fast, a slightly less accurate but much faster version of TIPP3, that is competitive in runtime with the other methods while being more accurate under challenging conditions.

In addition, we demonstrate that filtering input reads to only those that map to marker genes improves the profiling accuracy of Kraken2 [8], Bracken [9], and Metabuli [10] under most conditions, but that TIPP3 maintains an accuracy advantage over these methods for challenging conditions. Overall, we demonstrate that TIPP3 and TIPP3-fast are two valuable new additions to abundance profiling tools.

## Materials and methods

Here, we describe the materials and methods used in our study; for additional details, see Sects A–D in S1 Appendix.

### The TIPP pipeline

**Fig 1:**
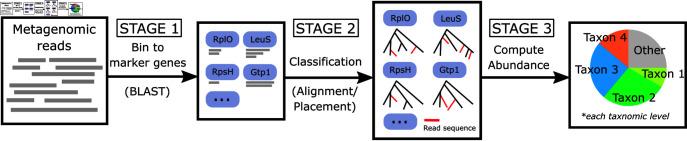
Overview of the TIPP pipeline. TIPP3 follows the same pipeline structure as TIPP and TIPP2 but differs in how some steps are performed in order to achieve higher accuracy and scalability. The common pipeline structure has three stages. Stage 1: Metagenomic reads are first binned to marker genes with BLAST. Stage 2: The query reads are added to the selected marker gene’s multiple sequence alignment, and a phylogenetic placement method is used to place reads into corresponding taxonomic trees using these alignments. Stage 3: Taxonomic labels are inferred from the placements and aggregated for the final abundance profile computation.

TIPP3 and its fast version, TIPP3-fast, both use the same basic pipeline structure as TIPP2 [17], but differ in how the specific steps are performed in order to obtain improved accuracy and computational performance. We begin with a high-level description of the common pipeline structure (see Fig 1), and then describe how TIPP3 differs from TIPP2.

Prior to running the method, a reference package consisting of a large set of marker genes with both alignments and taxonomic trees is constructed. The sequences from the marker genes are aggregated together to create a BLAST database for binning reads. Input reads are binned to marker gene sequences in the reference package, with a threshold of at least 50bp coverage. Then, binned reads are added to their corresponding marker gene multiple sequence alignments (MSAs) and placed into marker gene taxonomic trees. The placement within the taxonomic tree specifies some (perhaps all) of the taxonomic labels for the read, but only taxonomic levels with placement support above the user-selected threshold are considered. For example, if a read has 80% support at the species level and 98% support at the genus level and we use a support threshold of 95%, the read will be classified only at the genus level and higher. Then, the classification results are aggregated to form the final abundance profile.

Using Experiment 1, we designed TIPP3 (see below); here we briefly explain how TIPP3 differs from TIPP2.

#### Stage 1: Read binning.

BLAST [26] is used to bin input reads to their corresponding marker gene sequences ( ≥ 50bp coverage). If a read does not map to any marker gene sequence, then it is discarded from further analysis.

#### Stage 2: Read classification.

This stage can be broken into two sub-stages. We first use WITCH [27], a new method for adding sequences into MSAs, to add reads to the marker gene MSAs that they map to. Then, the query sequences are added into the relevant taxonomic tree using an improved technique for running pplacer, where it is run with the taxtastic package [28] (pplacer-taxtastic), which allows it to place reads into large taxonomic trees (up to 100,000 leaves). We use a support value of 90% for pplacer-taxtastic and assign taxonomic labels only at those levels that achieve at least the corresponding support values.

#### Stage 3: Abundance profile computation.

After reads are placed and classified from Stage 2, an abundance profile can be computed by pooling all read classifications. The relative abundance is computed as the total number of reads classified within a taxon divided by the total number of reads classified.

### TIPP3 vs. TIPP2

TIPP3 uses the same high-level algorithmic structure and the same techniques for Stage 1, but differs in the reference package construction (which is a precursor to the pipeline) and in Stage 2 (taxonomic classification of those reads that map to marker genes). Here, we describe the differences.

#### Reference package construction.

TIPP3 utilizes an updated NCBI taxonomy [29] and a much larger reference package than TIPP2, increasing the number of sequences per marker gene from  ∼ 4300 for TIPP2 to more than 50,000 for TIPP3. TIPP2 used PASTA [30] to compute the marker gene alignments, but TIPP3 uses MAGUS [31], which is a more accurate multiple sequence alignment method. MAGUS and PASTA both use a divide-and-conquer approach for aligning subsets of sequences, but MAGUS uses a more sophisticated technique for merging subset alignments compared to PASTA and produces more accurate multiple sequence alignments. TIPP2 used RAxML [32] to compute the taxonomic trees for their marker gene alignments, and TIPP3 uses RAxML-ng [33], but both use the NCBI taxonomy as the constraint tree.

#### Aligning and placing reads to marker genes.

While both TIPP2 and TIPP3 use BLAST to bin reads to marker genes, they use different techniques to add the reads to the marker gene MSAs and taxonomic trees. TIPP2 uses UPP [34] to add reads to the MSAs, and TIPP3 uses WITCH [27], which is more accurate. WITCH and UPP are two methods for adding sequences into a multiple sequence alignment; they are similar in their initial algorithmic design, in that they both represent the marker gene MSA using an ensemble of hidden Markov Models (HMMs), but then they differ in how the ensemble is used to add each query sequence. In UPP, each query sequence picks a single HMM in the ensemble based on the bit score, and the alignment of the query sequence to the single HMM then determines how the query sequence is added to the marker gene alignment. WITCH, in contrast, lets each query sequence pick the top few HMMs in the ensemble based on a corrected bit score, and then combines the resultant MSAs into a single MSA using a weighted ensemble approach. As shown in [27], the WITCH approach produces more accurate alignments than the UPP approach.

TIPP2 used pplacer for phylogenetic placement with RAxML numeric parameters, and TIPP3 uses new phylogenetic placement methods that can scale to larger reference trees. Specifically, TIPP3 uses pplacer-taxtastic [21], which uses the Python package taxtastic [28] for the numeric parameters; interestingly, this allows pplacer to place into trees with  ∼ 100,000 leaves [21].

### Abundance profiling methods

We developed a fast version of TIPP3 that we call TIPP3-fast, as described below. We compared TIPP3 to TIPP3-fast, Bracken [9], Kraken2 [8], mOTUsv3 [35], MetaPhlAn4 [14], and Metabuli [10] for abundance profiling accuracy on our testing data. Kraken2 is designed for sequence classification, and Bracken is intended to build abundance profiles based on Kraken2 outputs. Kraken2 is kmer-based and uses a large pre-built database to map reads to their lowest common ancestor taxon [7]. Bracken uses the output from Kraken2 classification and information about genomes in the database to estimate abundance at the species level and above. mOTUsv3 is a marker gene-based abundance profiling method and maps metagenomic reads to their corresponding marker gene cluster units in its database [15]. mOTUsv3 is designed for short reads, and needs data pre-processing to deal with long reads. As recommended in the mOTUs GitHub page, we used the “long read” option provided in mOTUs (starting with version 2) to break each long read into multiple short read segments and used the generated mock short reads for abundance profiling. Since MetaPhlAn4 uses a marker gene database different from the one used by TIPP3, we generated three separate testing datasets to compare TIPP3 to MetaPhlAn4, with known, mixed, and novel genomes for both.

Metabuli provides a custom script to create its database using user-defined genomes. Using this, we generated a custom Metabuli database with the same genomes used by TIPP3. MetaPhyler [11] was not included in this study because it is no longer actively developed or maintained.

Additionally, we evaluated modifications to TIPP3 obtained by replacing its maximum likelihood phylogenetic placement method by either APPLES-2 [23] or App-SpaM [36]. APPLES-2 is a distance-based method that can place sequences into very large trees (up to 200,000 leaves) [23]. App-SpaM is an alignment-free placement method designed for placing short sequences into an existing tree, based on their phylogenetic distances to sequences in the tree [36].

### Datasets and read simulation

For the training experiment, we used two TIPP2 datasets, one with 51 genomes and the other with 33 genomes (i.e., “Training” datasets). For the TIPP2 study, the 33 genomes were novel (i.e., not in the TIPP2 reference package) and the 51 genomes were known (i.e., in the TIPP2 reference package). The TIPP3 reference package contains all the genomes from the TIPP2 reference package as well as others. Therefore, this means that the 51 genomes are guaranteed to be known to TIPP3, but some of the 33 genomes might also be known. Of the 33 genomes that were novel to TIPP2, 19 are now “known” to TIPP3. Thus, the 33 genome case is now a combination of known and novel, and so is “mixed".

For testing, we created three mock microbial communities, denoted as “known”, “mixed”, and “novel” based on whether the genomes of the included species are present in the TIPP3 reference package. The “known” community has 50 known genomes, the “mixed” community has 53 known and 47 novel genomes, the “novel” community has 50 novel genomes, and the genomes of the three communities are disjoint. These mock communities are referred to as “Testing-1” in the following sections.

For a fair comparison to Bracken and Kraken2, we ensured that known genomes are also present in the Bracken/Kraken2 database. When a genome is novel to TIPP3, it is also not present in the Bracken/Kraken2 database. We used the “PlusPF” Kraken2 database published in June 2023 [37], which has the closest date to the NCBI taxonomy used for TIPP3.

When comparing to MetaPhlAn4, we selected a subset of genomes from each community, denoted as “Testing-2”, to ensure that known genomes are known to both TIPP3 and MetaPhlAn4, and novel ones are novel to both methods. Datasets from Testing-2 are only used for the comparison between TIPP3, TIPP3-fast, and MetaPhlAn4. We used the “vOct23” reference package updated in August 2024 of MetaPhlAn4.

We also evaluated TIPP3 on replicate 1 of the CAMI-II Marine dataset [38], with both short Illumina and long PacBio reads. To evaluate a method’s accuracy on this dataset, we have extracted the relative abundances directly from the abundances of the genomes used to construct the datasets. Links to the CAMI-II datasets can be found in Sect C in S1 Appendix.

#### Read simulation.

We simulated Illumina, PacBio, and Nanopore reads using ART sequence simulator [39], PBSIM [40], and NanoSim [41]. For training datasets, we only simulated Illumina and PacBio reads. For the Testing-2 datasets, which are used to evaluate MetaPhlAn4, we only simulated Illumina reads as MetaPhlAn4 is not suitable for long reads [42]. We show the properties of the simulated reads for each dataset in Table 1, and more details for read simulation can be found in Sect C in S1 Appendix.

### Evaluation criteria

**Normalized Hellinger distance.** The studies presenting TIPP [16] and TIPP2 [17] used the Hellinger distance [43] to measure the abundance profiling error of methods, defined as follows. Given a set of reads, the Hellinger distance of an estimated abundance profile to the true abundance profile on a taxonomic level (e.g., at the species level) is given by:


$$H_{l}=\frac{\sqrt{\underset{x\in C_{l}}{\sum }{\left(\sqrt{T_{x}}-\sqrt{E_{x}}\right)}^{2}}}{\sqrt{2}},$$

where $T_{x}$ is the true abundance and $E_{x}$ the estimated abundance of a clade *x*, for each *x* in the set of clades $C_{l}$ on a taxonomic level *l*. Reads that are unclassified at a certain level are not counted for the Hellinger distance calculation.

However, in certain cases, $H_{l}$ does not correctly reflect the actual profiling error of a method. Here, we present a new measurement, **Normalized Hellinger distance**, $H_{l}^{*}$, that provides unbiased measurements of estimated profiles in all cases. New variables included in the modified equation are *n*, the total number of reads classified, and $n_{l}$, the number of reads classified at taxonomic level *l*. See Sect D in S1 Appendix for the full derivation of the normalized Hellinger distance and an example of when Hellinger distance is unsuited.


$$\begin{array}{llll} H_{l}^{*} & =\frac{\sqrt{\underset{x\in C_{l}}{\sum }{\left(\sqrt{T_{x}}-\sqrt{E_{x}}\right)}^{2}}}{\sqrt{1+\frac{n_{l}}{n}}}\; & & \; \end{array}$$

#### Computational performance.

We also measure the wall-clock running time and maximum memory usage. Each method is run on the University of Illinois Campus Cluster given 16 CPU cores and 256 GB of memory.

## Results

**Table 1:** Properties of simulated reads for training and testing datasets.

Designation	Dataset	Type	Known/ Novel	Number of genomes	Number of reads	Mean length
Training	TIPP2-33	Illumina	19/14	33	10,026,239	150
	TIPP2-33	PacBio	19/14	33	1,001,875	3002
	TIPP2-51	Illumina	51/0	51	10,840,270	150
	TIPP2-51	PacBio	51/0	51	1,082,858	3003
Testing-1	Known-50	Illumina	50/0	50	10,500,910	150
	Known-50	PacBio	50/0	50	1,047,884	3006
	Known-50	Nanopore	50/0	50	184,327	4033
	Mixed-100	Illumina	53/47	100	26,303,844	150
	Mixed-100	PacBio	53/47	100	2,631,356	3004
	Mixed-100	Nanopore	53/47	100	368,653	4028
	Novel-50	Illumina	0/50	50	13,770,513	150
	Novel-50	PacBio	0/50	50	1,375,264	3004
	Novel-50	Nanopore	0/50	50	184,327	4028
Testing-2 (for MetaPhlAn4)	Known-25	Illumina	25/0	25	5,254,840	150
	Mixed-44	Illumina	22/22	44	11,056,728	150
	Novel-22	Illumina	0/22	22	5,424,010	150
Testing-3	CAMI-II-Marine	Illumina	-	476	33,301,262	150
	CAMI-II-Marine	PacBio	-	476	1,641,591	2968

### Overview

We include three experiments in this study.

1. In Experiment 1, we use the training data to set the algorithmic parameters for TIPP3, which include the alignment and phylogenetic placement methods for our binned query reads, and the set of marker genes that are used to filter reads for abundance profiling.
2. In Experiment 2, we evaluate the impact of filtering reads using the TIPP3 marker genes using the Testing-1 datasets.
3. In Experiment 3, we compare TIPP3 to TIPP2 and other leading abundance profiling methods: Bracken, Kraken2, mOTUsv3, MetaPhlAn4, and Metabuli, some with filtered reads, using all testing datasets.

### Experiment 1: Designing TIPP3

#### TIPP3 algorithmic parameters.

In this experiment, we explored different ways to run TIPP3 and decided on the most suitable TIPP3 pipeline optimizing for profiling accuracy and runtime. We explored (1) different ways to add reads to marker gene MSAs, (2) different ways to place reads into the marker gene taxonomic tree, and (3) different selections of marker genes for the aggregated abundance profile. We used the training datasets for this experiment. Here, we provide a summary of the results that determined the parameters for TIPP3; detailed experimental results can be found in Figs A–N and Sect E in S1 Appendix.

##### TIPP3.

The results show that when there are noticeable differences between the six variants, the biggest differences are due to the choice of alignment method, with the most accurate methods using WITCH instead of BLAST to add reads into the marker gene alignment. Thus, the alignment step is very important. We also saw that while no single placement method provided better accuracy than the others under all conditions, pplacer using the taxtastic package [28] (i.e., pplacer-taxtastic) had a slight advantage over the other placement methods. Based on the training results, we selected WITCH for adding reads to marker gene MSAs and pplacer-taxtastic for performing phylogenetic placement. Finally, we chose 38 marker genes (excluding FtsY and RpoB because of poor individual profiling results) for the aggregated abundance profile.

##### TIPP3-fast.

When optimizing for accuracy on our training data, we observed two bottlenecks for runtime. The first was the time used to add reads to marker gene alignments using the most accurate method tested, WITCH. The second was the read placement time required by pplacer-taxtastic, the most accurate method tested for placing reads into taxonomic trees of marker genes.

We performed a sequence of experiments on the training datasets to develop a variant of TIPP3 that would be fast and almost as accurate. We looked at two ways of adding query sequences into the marker gene alignment: WITCH and BLAST, and three ways of performing taxonomic placement: pplacer using taxtastic (as used in TIPP3), SCAMPP, and BSCAMPP. Thus, we compared five new pipelines to TIPP3.

Although our experiments revealed that adding reads into alignments using WITCH produced the most accurate results, we also saw that this choice had a very large impact on runtime; therefore we selected BLAST for the alignment step. These experiments also showed that pplacer-taxtastic was overall slightly more accurate than the other placement methods, the fastest of the three was BSCAMPP. Therefore, we selected BLAST for the alignment step and BSCAMPP for the placement step for the fast variant of TIPP3, and refer to this combination as TIPP3-fast.

### Experiment 2: Restricting abundance profiling methods to filtered reads

Kraken2, Bracken, and Metabuli are three methods we explored that are not based on any kind of marker gene analysis, unlike TIPP3, TIPP3-fast, MetaPhlAn4, and mOTUsv3. Here, we examined the impact of restricting these abundance profiling methods to just those reads that map to the marker genes from the TIPP3 reference package (i.e., filtering the input reads). We refer to these two different ways of running each method by appending either “(all)" or “(filtered)" to the method’s name. For this experiment, we used the Testing-1 datasets.

We explored this question and found that filtering improved accuracy for all three methods. Here, we show the results for Kraken2 and Bracken, but see Fig O and Sect F in S1 Appendix for results on Metabuli.

**Fig 2:**
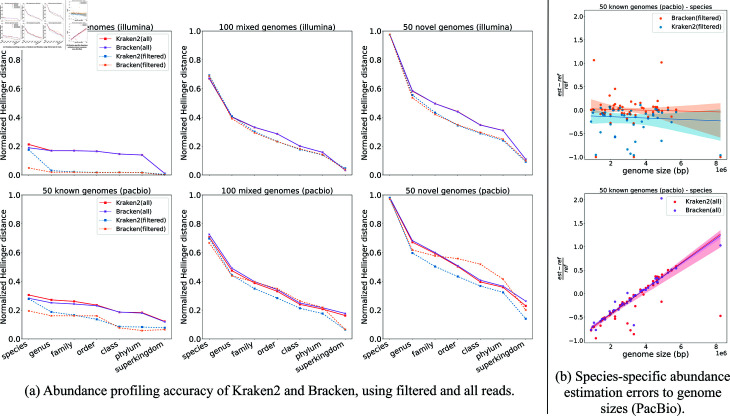
The impact of filtering reads on Kraken2 and Bracken for abundance profiling accuracy. (a) Abundance profiling accuracy by normalized Hellinger distance (lower means more accurate) of two ways of running Kraken2 and Bracken on Illumina and PacBio reads from three mock microbial communities (50 known, 100 mixed, and 50 novel genomes). Dashed lines correspond to using filtered reads, and solid lines correspond to using all (unfiltered) reads. (b) Scatter plot of species-specific abundance estimation errors (PacBio reads) to corresponding genome sizes for 50 known genomes of Bracken and Kraken2 using filtered or all reads as inputs. The estimation error for each taxon is calculated as the fractional difference between its estimated abundance and the reference abundance (y-axis). A Robust Linear Model with Huber Loss [44] was used to fit a regression line for each method. The shaded area around each fitted line represents a 95% confidence interval of the corresponding method.

Fig 2(a) shows the impact on profiling accuracy for filtering Kraken2 and Bracken. Filtering consistently improves accuracy for both Kraken2 and Bracken when working with Illumina reads. For PacBio reads, filtering continues to enhance Kraken2’s accuracy, but this isn’t always true for Bracken. While Bracken(filtered) outperforms Bracken(all) at the species and genus levels for PacBio reads, in some cases, it increases profiling errors compared to the unfiltered version. This issue is particularly noticeable at the order, class, and phylum levels when profiling reads from novel genomes, where Bracken(filtered) is less accurate than Bracken(all).

To better understand why filtering improves accuracy for Kraken2 and Bracken, we investigated the impact of genome size on abundance profiling error on PacBio reads, when using either all the reads or only those that map to the marker gene, taken from known genomes. We plotted the fractional estimation errors for individual species against their corresponding genome sizes and computed a Robust Linear Model with Huber Loss [44] to fit a regression line for each method, with a 95% confidence interval displayed. The results (Fig 2(b)) show that when using filtered reads, Bracken and Kraken2 exhibit estimation errors that are independent of genome sizes; however, when using all input reads there is a strong linear increase in error as the genome size increases.

In summary, we do see some conditions (specifically, PacBio reads from novel genomes) where filtering does not improve Bracken2 and can even reduce accuracy, but in general, filtering improves or maintains accuracy for all three methods – and consistently so at the species through family levels. Since the primary focus of abundance profiling is typically on lower taxonomic levels (species and genus) and filtering improves accuracy at these levels, we present results only for the filtered versions of these methods in the remaining figures.

### Experiment 3: Evaluation of TIPP3 for abundance profiling

#### Experiment 3a: Comparing TIPP3 to TIPP2.

Using our testing datasets, we now demonstrate the impact of using a larger reference package within the TIPP3 pipeline by comparing TIPP3 to “TIPP3-small”, a version of TIPP3 restricted to using a smaller reference package.

We generated a new reference package for TIPP3-small by sub-sampling the TIPP3 marker gene taxonomic trees and alignments, selecting 1-3 genomes per genus. This allows the taxonomy for each marker gene to contain  ∼ 5505 sequences, a factor of 10 reduction from the TIPP3 reference package ( ∼ 55,000 sequences per marker gene) but still larger than the TIPP2 reference package ( ∼ 4300 sequences in TIPP2).

**Fig 3:**
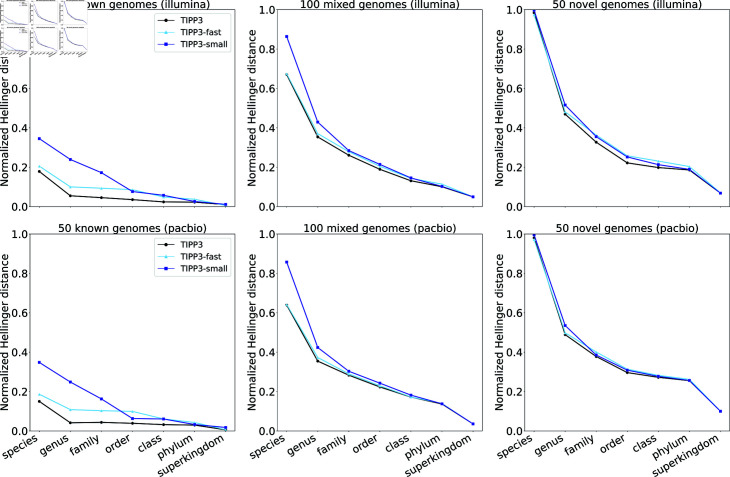
Normalized Hellinger distance of TIPP3, TIPP3-fast, and TIPP3-small profiling reads from mock microbial communities with known, mixed, and novel genomes. Both TIPP3 and TIPP3-small use WITCH to add query reads to marker gene MSAs, and TIPP3-fast uses BLAST to compute query read alignments to marker gene MSAs. TIPP3 uses pplacer with the taxtastic package for placement and a support value of 90%. TIPP3-fast uses BSCAMPP for placement and a support value of 95%. TIPP3-small uses pplacer for query placement and a support value of 95%, the same setup in TIPP2 [17].

Fig 3 compares TIPP3 and TIPP3-fast to TIPP3-small for abundance profiling accuracy on Illumina and PacBio reads from the Testing-1 datasets, using normalized Hellinger distance. For all six testing datasets, TIPP3 is consistently more accurate than TIPP3-small. The difference between the two methods is more noticeable on lower taxonomic levels such as species, genus, and family, particularly for reads from known genomes. TIPP3-fast is also generally more accurate than TIPP3-small, with a few exceptions where TIPP3-small is on par or even more accurate (e.g., order level for known PacBio reads). As we include more novel genomes in our dataset, errors in the three methods increase and their difference in profiling accuracy decreases, especially on the lower taxonomic levels such as species and genus. These trends are consistent across both Illumina- and PacBio-style reads, showing that TIPP3 and TIPP3-fast improve upon TIPP2 through a more densely sampled reference package.

#### Experiment 3b: Comparing TIPP3 to other methods on mock microbial communities.

We explored the impact of substituting the maximum likelihood-based phylogenetic placement methods (pplacer-taxtastic for TIPP3 and BSCAMPP for TIPP3-fast) by either a distance-based method (APPLES-2 [23]) or an alignment-free method (AppSpaM [36]). These experiments, shown in Fig P in S1 Appendix, establish that changing the phylogenetic placement method to either APPLES-2 or App-SpaM reduces accuracy.

**Fig 4:**
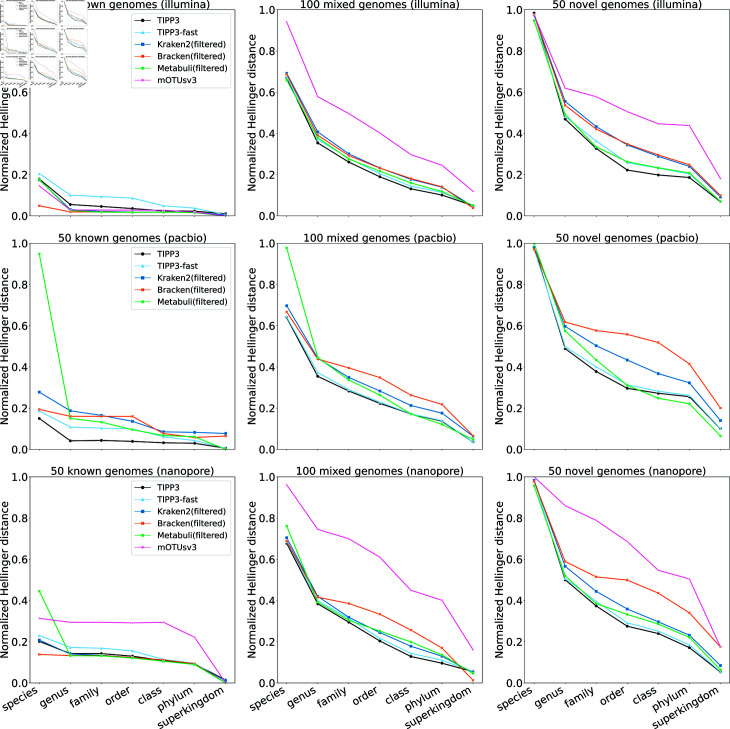
Normalized Hellinger distance of methods profiling reads from mock microbial communities with known, mixed, and novel genomes. For PacBio read datasets, mOTUsv3 did not produce any classification or profile and thus is absent.

A comparison of TIPP3 and TIPP3-fast to the other methods is shown in Fig 4. We note that mOTUsv3 did not output any classification for any PacBio reads, even using the pre-processing step recommended by the authors of mOTUsv3 to deal with long reads (a strategy that is also used in [45]).

##### Summary of trends.

These experiments establish that the relative and absolute accuracy depends on the sequencing technology, whether the reads are known, mixed, or novel, and the taxonomic level. Error rates for all methods are lowest for Illumina short reads, and then higher for PacBio and Nanopore long reads. Error rates for all methods are also lowest for reads from known genomes, higher for the mixed case where reads are from known and novel genomes and highest for entirely novel genomes. All these trends are as expected.

The relative accuracy of methods is nevertheless dependent on the model condition. Under the easiest condition of Illumina reads from known genomes, error rates are low and differences are mostly noteworthy only at the species level. At that level, TIPP3-fast is the least accurate method, and TIPP3 ties with Metabuli(filtered) for the second least accurate. Bracken(filtered) is the most accurate, and all other methods are just slightly better than TIPP3.

For all other conditions, TIPP3 is typically the most accurate, TIPP3-fast is often the second most accurate, and the gap between TIPP3-fast and the next most accurate is often large. We also see that mOTUsv3 generally has poorer accuracy than the other methods, Metabuli(filtered) often has among the highest error of the tested methods at the species level but can be close to the most accurate at the higher taxonomic levels, and Bracken(filtered) is the most accurate at the species level for known genomes for both Illumina and Nanopore reads.

##### Comparing to MetaPhlAn4.

**Fig 5:**
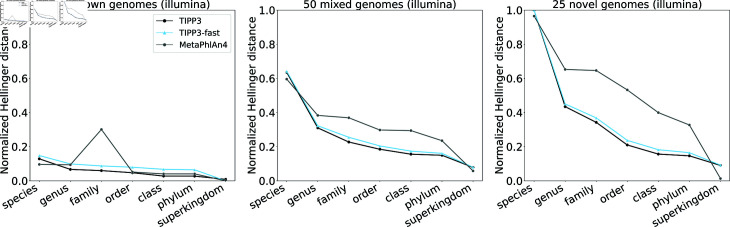
Normalized Hellinger distance of TIPP3, TIPP3-fast, and MetaPhlAn4 profiling Illumina reads from mock microbial communities with known, mixed, and novel genomes.

Fig 5 shows the comparisons between TIPP3, TIPP3-fast, and MetaPhlAn4 on Illumina reads from the Testing-2 datasets. We do not show results for PacBio or Nanopore reads because MetaPhlAn4 does not support analysis of long read sequences [42], which we confirmed when our initial attempt using MetaPhlAn4 on PacBio failed to return a profile.

The relative accuracy between TIPP3/TIPP3-fast and MetaPhlAn4 depends on the taxonomic levels and whether genomes are known, mixed, or novel. However, TIPP3 had a small but consistent advantage over TIPP3-fast at all settings, which is expected.

On reads from known genomes, MetaPhlAn4 was more accurate at the species and genus levels than TIPP3 and TIPP3-fast, but had a sudden increase in error at the family level, for which we do not have an explanation. At all other levels, TIPP ties with MetaPhlAn4 in profiling accuracy, and TIPP3-fast is slightly less accurate.

For mixed genomes, error rates increase for all methods but especially for MetaPhlAn4, so that MetaPhlAn4 has the highest error at the genus through phylum levels. MetaPhlAn4 is slightly more accurate than TIPP3 and TIPP3-fast at the species and superkingdom levels.

For novel genomes, error rates increase for all methods, and the gap between MetaPhlAn4 and TIPP3/TIPP3-fast again increases, with MetaPhlAn4 having higher error rates at the genus through phylum levels. MetaPhlAn4 has lower error than TIPP3/TIPP3-fast at the species and superkingdom levels.

#### Experiment 3c: Results on the CAMI-II dataset.

We evaluated TIPP3 and TIPP3-fast on the CAMI-II Marine dataset (replicate 1) with Illumina and PacBio reads [38] and compared their profiling accuracy to Kraken2(filtered), Bracken(filtered), Metabuli(filtered), mOTUsv3, and MetaPhlAn4.

**Fig 6:**
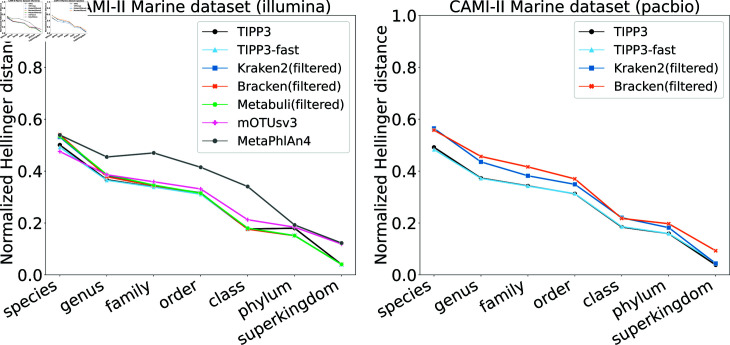
Normalized Hellinger distance of methods profiling Illumina and PacBio reads from the CAMI-II Marine dataset. Metabuli(filtered), mOTUsv3, and MetaPhlAn4 did not produce a profile for CAMI-II Marine PacBio reads.

For CAMI-II Marine Illumina reads, MetaPhlAn4 has the highest error of all methods; the remaining methods have very similar accuracy, with mOTUsv3 being the most accurate at the species level but less accurate otherwise. TIPP3 and TIPP3-fast are the second most accurate at the species level and the most accurate at the genus level (Fig 6).

For CAMI-II Marine PacBio reads, the comparison is between TIPP3, TIPP3-fast, Kraken2(filtered), and Bracken2(filtered), as the other methods did not produce a profile. TIPP3 and TIPP3-fast are the most accurate methods at all levels, and tie for most accurate at the superkingdom level. Bracken(filtered) and Kraken2(filtered) are very close, but with a small advantage to Kraken2(filtered).

#### Experiment 3d: Detailed evaluation on species abundances.

**Fig 7:**
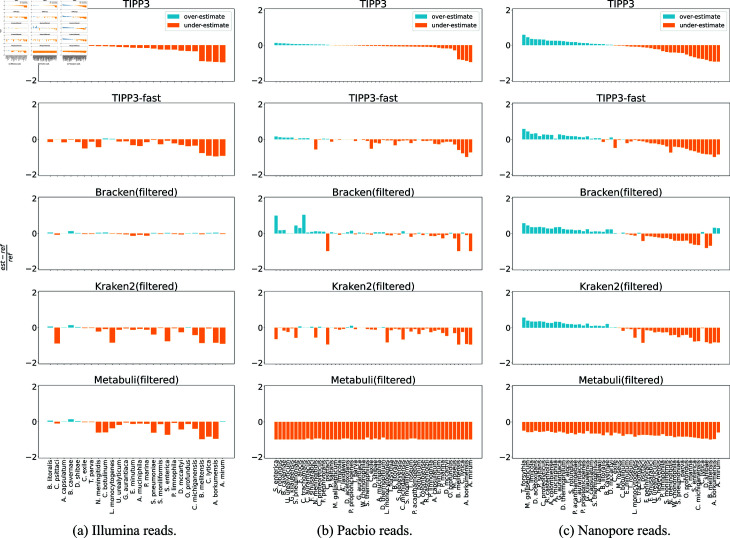
Species-specific abundance estimation error of methods profiling reads from a mock microbial community with known genomes. (a) Illumina reads. (b) PacBio reads. (c) Nanopore reads. mOTUsv3 is excluded because it either produced no profile or had high abundance profiling errors except for Illumina reads. The estimation error is shown on the y-axis. For each comparison, a taxon is shown if and only if it is present in the reference and at least one method has an estimation error strictly greater than 10% in magnitude. Species are sorted left-to-right by TIPP3’s error, from overestimation to underestimation. Full results for all datasets at species and genus levels can be found in Sect G in S1 Appendix.

To better understand what contributes to the profiling error of each method, we also examine abundance profiling error on a per-species basis. The estimation error is given by $\frac{est-ref}{ref}$ where *est* is the estimated abundance and *ref* is the true (reference) abundance of a species. We examined the mock communities with Illumina, PacBio, and Nanopore reads from 50 known genomes and selected the subset of species for which at least one of the top-performing methods (TIPP3, TIPP3-fast, Kraken2(filtered), Bracken(filtered), and Metabuli(filtered)) has an abundance profiling error greater than 10% above or below the correct value. Results are shown in Fig 7 and Figs Q–T in S1 Appendix.

##### Illumina reads of 50 known genomes.

Bracken(filtered) has nearly zero estimation error for these per-species abundance measurements (Fig 7(a)). TIPP3, TIPP3-fast, Kraken2(filtered), and Metabuli(filtered) display a similar error composition, generally having more underestimation errors compared to Bracken(filtered), while having few overestimation errors. These results are consistent with the relative performance shown in Fig 4.

##### PacBio reads of 50 known genomes.

For PacBio reads (Fig 7(b)), TIPP3 demonstrates the highest accuracy, followed closely by TIPP3-fast, with the primary source of error for both methods being underestimation. Similarly, Kraken2(filtered) tends to underestimate species abundances, consistent with the observations regarding Illumina reads. The comparison between Bracken(filtered) and Kraken2(filtered) is more complicated; Bracken(filtered) has more overestimation errors than Kraken2(filtered) but has much fewer underestimation errors. Interestingly, according to Fig 4, Bracken(filtered) has an overall lower abundance profiling error, using the normalized Hellinger distance, than Kraken2(filtered). On the other hand, Metabuli(filtered) produced a profile that underestimated many species abundances.

##### Nanopore reads of 50 known genomes.

Nanopore results show an interesting trend of over and underestimations that TIPP3, TIPP3-fast, Bracken(filtered), and Kraken2(filtered) shared (Fig 7(c)), except that Bracken(filtered) slightly overestimated the abundances of some species that the three methods above clearly underestimated. This difference in error profile could be the main contributor to why Bracken(filtered) has the highest profiling accuracy at the species level shown in Fig 4. Finally, Metabuli(filtered) again produced a profile that underestimates many species abundances.

#### Runtime and memory.

All methods were given 16 cores of CPU and 256 GB of memory and allowed to run to completion. We used the University of Illinois, Urbana-Champaign Campus Cluster, which is a heterogeneous runtime environment with a mixture of old and new generations of CPUs, making the runtime comparison somewhat unreliable. Given this caveat, we report runtime and memory usage until an output abundance profile was computed, including the runtime for filtering reads for some methods (TIPP3, TIPP3-fast, Bracken(filtered), Kraken(filtered), and Metabuli(filtered)). The runtime and memory of all methods, including Bracken, Kraken2, and Metabuli using all reads as input, are shown in Figs U and V and Table A in S1 Appendix.

**Fig 8:**
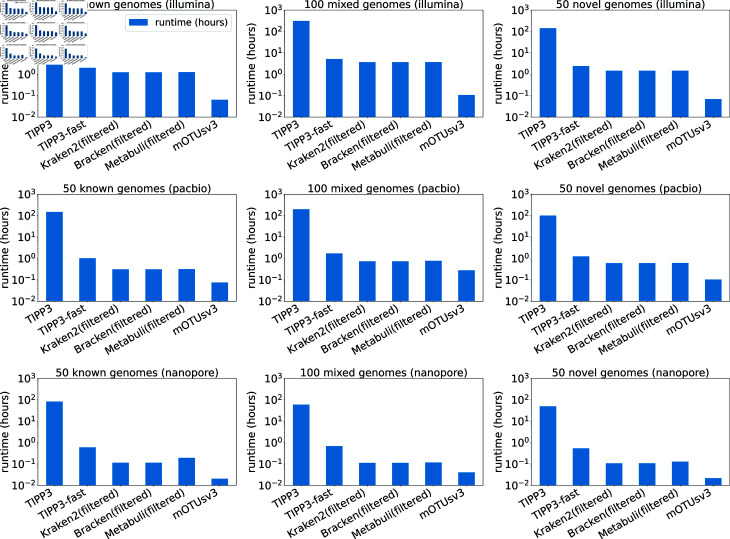
Runtimes of abundance profiling methods on Testing-1 datasets.

Fig 8 shows the runtime for TIPP3, TIPP3-fast, Kraken2(filtered), Bracken(filtered), Metabuli(filtered), and mOTUsv3 for the Testing-1 datasets. All methods were able to complete each dataset with no runtime error. On all datasets, TIPP3 required the longest time to complete (101–312 hours), with a large portion of the runtime dedicated to running WITCH to add query reads to marker gene MSAs. The other methods were able to complete each testing dataset in less than 6 hours. The fastest methods are Kraken2(filterd), Bracken(filtered), Metabuli(filtered), and mOTUsv3, taking 0.1–3.7 hours. TIPP3-fast is slightly slower at 0.5–5.2 hours, but on average achieved a 94x speedup compared to TIPP3 on the Testing-1 datasets (Table B in S1 Appendix).

Memory usage of methods is shown in Fig V in S1 Appendix. All methods tested were able to complete each testing dataset within the 256 GB limit. Peak memory usage for TIPP3 was among the highest alongside Kraken2(filtered) and Bracken(filtered). TIPP3-fast used less memory on all datasets than TIPP3 and under most conditions is one of the most memory-efficient, but we also found it to have high memory usage when the input size was large (e.g., Illumina reads from 100 mixed genomes). Metabuli(filtered) and mOTUsv3 are the other two memory-efficient methods, consistently using less than 32 GB of memory across all Testing-1 datasets. For Kraken2(filtered) and Bracken(filtered), the high memory usage was likely due to loading the database to memory. However, it is possible that the memory usage could be reduced substantially using the –memory-mapping option, as suggested in [8], which was not used in this study.

## Discussion

Our experiment revealed several consistent trends that are expected based on prior studies (e.g., [17]). For example, for all methods, abundance profiling error is higher for Nanopore and PacBio reads than Illumina reads and higher for novel genomes than for known genomes. Another consistently observed trend is that error goes down as the taxonomic level increases.

Our results generally showed that TIPP3 had superior accuracy compared to the other methods, except for the easiest condition (Illumina reads from known genomes), where other methods were more accurate at the lower taxonomic levels. However, on the more challenging datasets, where there was either sequencing error or the reads were partially or fully from novel genomes, TIPP3 had an advantage. TIPP3-fast was generally slightly less accurate than TIPP3 but much faster; in challenging conditions, it was often more accurate than the other abundance profiling methods. Thus, both TIPP3 and TIPP3-fast offer high accuracy, with TIPP3 somewhat more accurate than TIPP3-fast.

We now consider the design elements that contribute to the accuracy advantage of TPP3 and TIPP3-fast over the other methods, including TIPP2, for these more challenging conditions. One design aspect is the restriction of the reads to marker genes, which is a characteristic of all methods based on marker genes. Our study shows clearly that restricting the input reads to TIPP3 marker genes improves accuracy for Kraken2, Bracken, and Metabuli, thus strongly supporting that this is an important part of TIPP3’s accuracy.

But TIPP3 differs from many other methods that use marker genes by its algorithmic structure: it performs phylogenetic placement of aligned reads into taxonomies. In Experiment 1, we showed that modifications to TIPP3’s design so that it uses other phylogenetic placement methods or other techniques to align reads reduce accuracy. Indeed, TIPP3-fast changes each of those steps in order to improve speed, and has lower accuracy than TIPP3 for challenging conditions. Hence, how these steps are implemented is important.

Our study demonstrated that part of the reason TIPP3 has high accuracy is its use of larger marker gene-based taxonomies in its reference package. Given the substantial improvement in accuracy, this shows the benefit of using more densely sampled taxonomic trees for abundance profiling. This observation is consistent with the improvement of TIPP2 relative to TIPP [17]. Furthermore, this is also consistent with prior studies that have shown that more accurate phylogenetic placement can be obtained through the use of larger and more densely sampled reference trees [24,25].

Finally, our study demonstrated the importance of choosing marker genes carefully: although we started with 40 marker genes, we selected only 38 of them for use in TIPP because this change improved accuracy. Our exploration of having a further reduction in the number of genes showed a reduction in accuracy, without a substantial improvement in runtime, and was discarded (Figs W and X in S1 Appendix). Thus, the choice of marker genes to include has an impact on accuracy.

## Conclusions

In this study, we introduced a new method, TIPP3, for accurate abundance profiling. TIPP3 outperforms its predecessor TIPP2 in terms of profiling accuracy and also provides more accurate profiles than other taxonomic profiling tools when input reads have sequencing errors and come from genomes absent from reference databases used by these tools. TIPP3-fast is a much faster version of TIPP3, having a runtime comparable to Kraken2, Bracken, Metabuli, and mOTUs and with only a small decrease in accuracy compared to TIPP3. Therefore, TIPP3-fast maintains TIPP3’s accuracy advantage over the other methods under conditions that are challenging for abundance profiling. Given that microbial communities are abundant but mostly still under-explored and may include many currently unknown genomes, tools such as TIPP3 and TIPP3-fast are valuable for the accurate characterization of these microbial communities.

One of the reasons that TIPP3 provides high accuracy is that it is based on filtering the reads to a selected set of marker genes, which are genes that appear universally and are single copy. This study showed that filtering abundance profiling methods to the marker gene set of TIPP3 generally improved their profiling accuracy, sometimes very substantially. Thus, when abundance profiling is the objective, restricting the input to marker genes is potentially highly beneficial.

Based on this study, we can make some recommendations for the choice of abundance profiling method. When working with Illumina reads from known genomes, then Bracken(filtered) is the most accurate method (and much more accurate than Bracken(all)). However, for other conditions, then TIPP3 is the most accurate, followed by TIPP3-fast. The choice between TIPP3 and TIPP3-fast essentially depends on how important runtime is compared to accuracy, as TIPP3 is much slower (50 to 150 times slower) than TIPP3-fast.

This study suggests several directions for further improvement. While TIPP3 achieves high profiling accuracy using the most accurate setting, it has a significantly slower runtime compared to other methods. The step in TIPP3 where reads are added into the marker gene alignment using WITCH is the biggest contribution to runtime, which suggests that developing new methods for this step that are substantially faster but not much less accurate than WITCH is a promising direction.

Another direction for future research is algorithm design to enable accuracy to continue to improve as the number of sequences in each marker gene increases. Although most of the algorithmic steps in TIPP3 are already known to work well on very large datasets (e.g., MAGUS for the marker gene alignment and WITCH for adding reads to marker gene alignments), pplacer-taxtastic is possibly restricted to about 100,000 sequences. If so, then either we would need to improve the scalability of pplacer-taxtastic, or rely on BSCAMPP and possibly other fast methods for phylogenetic placement.

## Supporting information

S1 Appendix All supporting text, 24 supporting figures, and 2 supporting tables are included. (PDF)
