# Supplementary material for: TIPP3 and TIPP3-fast: Improved abundance profiling in metagenomics
Source: PLoS Comput Biol. 2025 Apr 4;21(4):e1012593. doi: 10.1371/journal.pcbi.1012593 (PMC11970662; doi:10.1371/journal.pcbi.1012593)
Supplement: S1 Appendix — (PDF) [file pcbi.1012593.s001.pdf]

Appendix for "TIPP3 and TIPP3-fast: Improved Abundance Profiling in Metagenomics"

Chengze Shen[1], Eleanor Wedell[1], Mihai Pop[2], Tandy Warnow[1*],

1 Siebel School of Computing and Data Science, University of Illinois Urbana-Champaign, Urbana, IL, US
2 Department of Computer Science, University of Maryland at College Park, College Park, MD, US

* warnow@illinois.edu

Contents

A Additional Information on TIPP3 Reference Packages 3

B Software Commands 5

C Additional Information for Dataset and Read Simulation 7

D Additional Information for Normalized Hellinger Distance 10
D.1 Normalized Hellinger distance . . . 10
D.2 Example for biases with Hellinger distance . . . 11

E Additional Results for Experiment 1: Designing TIPP3 12
E.1 Adding reads to marker gene alignments . . . 12
E.2 Placing reads into marker gene taxonomies . . . 16
E.2.1 BSCAMPP . . . 17
E.2.2 SCAMPP . . . 19
E.2.3 pplacer-taxtastic . . . 21
E.2.4 Comparison of the three placement methods . . . 23
E.3 Marker gene selection . . . 25
E.3.1 Abundance profile on each marker gene . . . 26
E.3.2 Abundance profile using different sets of marker genes . . . 27

F Additional Results for Experiment 2: Restricting methods to filtered reads 28

G Additional Results for Experiment 3: Evaluation of TIPP3 for abundance profiling 29
G.1 Abundance profile comparison for all methods . . . 29
G.2 Detailed evaluation on species and genus abundances . . . 30
G.3 Runtime and memory usage . . . 34
G.4 Improving TIPP3 runtime performance . . . 38

List of Figures

A Experiment 1 (Training): taxonomic identification accuracy, UPP vs. WITCH, lower taxonomic levels . . . 13
B Experiment 1 (Training): taxonomic identification accuracy, UPP vs. WITCH, higher taxonomic levels . . . 14
C Experiment 1 (Training): runtime and memory usage, UPP vs. WITCH 15
D Experiment 1 (Training): taxonomic identification accuracy, BSCAMPP variants, lower taxonomic levels . . . 17
E Experiment 1 (Training): abundance profile accuracy, BSCAMPP variants 18
F Experiment 1 (Training): taxonomic identification accuracy, SCAMPP variants, lower taxonomic levels . . . 19
G Experiment 1 (Training): abundance profile accuracy, SCAMPP variants 20
H Experiment 1 (Training): taxonomic identification accuracy, pplacer-taxtastic variants, lower taxonomic levels . . . 21
I Experiment 1 (Training): abundance profile accuracy, pplacer-taxtastic variants . . . 22
J Experiment 1 (Training): abundance profile accuracy, best variants comparison . . . 23
K Experiment 1 (Training): runtime and memory usage, best variants comparison (WITCH alignment) . . . 24
L Experiment 1 (Training): runtime and memory usage, best variants comparison (BLAST alignment) . . . 25
M Experiment 1 (Training): abundance profile accuracy, individual marker genes . . . 26
N Experiment 1 (Training): abundance profile accuracy, different sets of marker genes . . . 27
O Experiment 2 (testing): abundance profile accuracy, Metabuli filtered and all reads . . . 28
P Experiment 3 (Testing): abundance profile accuracy, all methods . . . 29
Q Experiment 3 (Testing): abundance estimation error, 50 known genomes, genus . . . 30
R Experiment 3 (Testing): abundance estimation error, 100 mixed genomes, species . . . 31
S Experiment 3 (Testing): abundance estimation error, 100 mixed genomes, genus . . . 32
T Experiment 3 (Testing): abundance estimation error, 50 novel genomes, genus . . . 33
U Experiment 3 (Testing): runtime usage, all methods . . . 34
V Experiment 3 (Testing): memory usage, all methods . . . 35
W Abundance profile accuracy, TIPP3 variants . . . 39
X Runtime and memory usage, TIPP3 variants . . . 40

List of Tables

A Experiment 3 (Testing): All methods runtime in hours . . . 36
B Experiment 3 (Testing): TIPP3-fast vs. TIPP3 runtime speedup . . . 37

This document provides the supporting materials for the paper about TIPP3, including additional information for obtaining the reference package, software commands for benchmarking, information for input read simulation and normalized Hellinger distance, and additional results.

A Additional Information on TIPP3 Reference Packages

We used the same NCBI RefSeq Bacteria and Archaea genomes as TIPP2 [1], downloaded in November 2019 from the NCBI RefSeq database. The TIPP3 reference package was then constructed using the following pipeline. We first extracted and cleaned the same set of 40 marker genes as TIPP2 from the RefSeq genomes. After filtering, we aligned each set of marker gene sequences with MAGUS [2] and built a taxonomy tree with RAxML [3]. For running pplacer [4] with the taxtastic package [5], we re-estimated tree branch lengths with FastTree-2 [6]. The latest TIPP3 reference package is available at `https://doi.org/10.13012/B2IDB-4931852_V1`. Scripts for data processing can be found at `https://github.com/shahnidhi/TIPP_reference_package`.

Pre-alignment data processing

1. Filter the set of Archaea and Bacteria genomes (a total of 173,240 genomes) for each marker gene by (1) removing sequences that do not have matched amino/nucleotide sequences, (2) removing sequences that are 3 standard deviations from the median length. (with `data/filterData.py`).

2. Update the taxids using the **NCBI taxonomy downloaded on July 5th, 2023** (with `taxit update_taxids`).

3. Create a new taxonomy table for each marker gene (with `taxit taxtable`).

4. Update species mapping (species name to taxid) (with `update_species_mapping.py`).

5. Build taxonomy for each marker gene (with `build_unrefined_tree.pl`).

6. The outputs, for each marker gene, are (1) a final filtered set of sequences ($\sim 55,000$ sequences), and (2) an unrefined taxonomy of the sequences.

Alignment

1. Perform a non-recursive MAGUS alignment [2] on the filtered sequences for each marker gene.

2. The exact command of non-recursive MAGUS:

```
$ magus.py -np 16 -i [filtered unaligned sequences] \
    --recurse false \
    -d [output directory] -o [output alignment path]
```

3. The output is an alignment for each set of marker gene sequences.

Build taxonomy

1. Back-translate the amino acid alignment to nucleotide alignment (with `backtranslate_refseq.py`).

2. Clean up the tree internal labels on the taxonomy before refining it (with `nw_topology -bI`).

3. Mask gappy sites (> 95% gaps) of the nucleotide alignment (with `ogcat mask -p 0.95`).

4. Resolve polytomies with RAxML [3] under the GTR+CAT model.

```
$ raxml-PTHREADS-AVX -s [masked alignment] -m GTRCAT \
    -n [output prefix] -g [cleaned taxonomy] \
    -T 24 -p 12345 -w [work directory]
```

5. Add branch length information to the RAxML refined tree.

```
$ raxml-PTHREADS-AVX -s [masked alignment] -m GTRCAT \
    -n [output prefix] -F -f e [RAxML refined tree] \
    -T 24 -p 54321 -w [work directory]
```

6. Re-estimate the branch lengths with RAxML-ng [7] under the GTR+GAMMA model.

```
$ raxml-ng --evaluate --msa [masked alignment] \
    --model GTR+G --tree [RAxML branch-length tree] \
    --brlen scaled --redo --force perf_threads \
    --prefix [output prefix] --threads 24
```

7. Add back the internal labels with taxonomic information (with `relabel-modified.py`).

Re-estimating numeric parameters For `pplacer` [4] running with the `taxtastic` Python package [5], we re-estimated the taxonomy's numeric parameters with FastTree-2 [6] using the following command:

```
$ FastTreeMP --nosupport -gtr -gamma -nt -mllen -nome \
    -log [FastTree-2 log] -intree [inferred taxonomy] \
    < [masked alignment] > [re-estimated taxonomy]
```

Then, a `taxtastic` package is created as:

```
$ taxit create -P [output package dir] -l [name] \
    --aln-fast [masked alignment] \
    --tree-file [re-estimated taxonomy] \
    --tree-stats [FastTree-2 log]
```

B Software Commands

We ran all software with 16 CPU cores and up to 256 GB of memory, running each software with no time limit until completion.

1. We ran Kraken2 (v2.1.3) with the following command:

```
$ kraken2 --db [Kraken2 database] \
    --threads 16 --report [Kraken2 report] \
    [query reads file] > [Kraken2 output]
```

2. We ran Bracken (v2.9) with the following command (using Kraken2 output):

```
$ bracken -d [Bracken database] -i [Kraken2 report] \
    -w [Bracken report] -t 10 -r 150 \
    -o [Bracken output]
```

3. We ran mOTUsv3 (v3.1.0) with the following command:

```
$ motus profile -db [mOTUs database] \
    -q -u -p -c -s [query reads file] \
    -CC 16 -t 16 -o [mOTUs output]
```

4. We ran MetaPhlAn4 (v4.1.1) with the following command, using its "vOct23" database updated on August 2024.

```
$ metaphlan --nproc 16 --bt2_ps very-sensitive \
    --bowtie2db [metaphlan database] \
    --index [database name] \
    --bowtie2out [bowtie2 output] \
    -o [metaphlan output] --input_type fasta \
    [query reads file]
```

5. We ran Metabuli (v1.0.8) with the following command (when dealing with short Illumina reads):

```
$ metabuli classify [query reads file] \
    [metabuli database] [metabuli outdir] \
    [job ID] --seq-mode 1 --min-score 0.15 \
    --min-sp-score 0.5 \
    --threads 16 --max-ram 128
```

When dealing with long reads such as PacBio and Nanopore, we used the following command:

```
$ metabuli classify [query reads file] \
    [metabuli database] [metabuli outdir] \
    [job ID] --seq-mode 3 --min-score 0.07 \
    --min-sp-score 0.3 \
    --threads 16 --max-ram 128
```

6. We ran APPLES-2 (v2.0.11) with the following command (for each marker gene and its assigned reads):

```
$ run_apples.py -t [marker gene taxonomy] \
    -s [marker gene alignment] -q [query reads file] \
    -T 16 -o [placement results] -X -D
```

7. We ran App-SpaM (v1.03) with the following command (for each marker gene and its assigned reads):

```
$ appspam -t [marker gene taxonomy] \
    -s [marker gene sequences] -q [query reads file] \
    --threads 16 -o [placement results]
```

8. We ran SCAMPP (v2.0.1) with the following command (for each marker gene and its assigned reads):

```
$ pplacer-SCAMPP.py -b [subtree size] \
    -i [marker gene taxonomy RAxML info] \
    -t [marker gene taxonomy] -d [output dir] \
    -o [placement results] -a [query read alignment] \
    --threads 16
```

9. We ran BSCAMPP (v1.0.0) with the following command (for each marker gene and its assigned reads):

```
$ EPA-ng-BSCAMPP.py -b [subtree size] \
    -i [marker gene taxonomy RAxML info] \
    -t [marker gene taxonomy] -d [output dir] \
    -o [placement results] -a [query read alignment] \
    --threads 16
```

10. We ran pplacer (v1.1.alpha19-0-g807f6f3) with the `taxtastic` (v0.10.0) package with the following command (for each marker gene and its assigned reads):

```
// re-estimating marker gene taxonomy branch lengths with FastTree-2
$ FastTreeMP -nosupport -gtr -gamma -nt \
    -mllen -nome -log [FastTree-2 log file] \
    -intree [marker gene taxonomy]

// getting taxtastic reference package
$ taxit create -P [taxtastic reference package] \
    -l [marker gene name] \
    --aln-fast [marker gene alignment] \
    --tree-file [FastTree-2 re-estimated tree] \
    --tree-stats [FastTree-2 log file]

// running pplacer
$ pplacer -m GTR -c [taxtastic reference package] \
    -o [placement results] -j 16 \
    [query read alignment]
```

C Additional Information for Dataset and Read Simulation

Reads are simulated for each set of reference genomes using the ART sequence simulator [8] for Illumina reads, PBSIM [9] for PacBio reads, or NanoSim [10] for Nanopore reads. The commands used are shown below.

Illumina reads simulation Illumina reads are simulated with `art_illumina` v2.5.8 with the following command. The sequence length is 150 bp and the coverage is 20x.

```
$ art_illumina -ss HS25 -sam \
     -i [genome path] -p -l 150 -f 20 -m 200 \
     -s 10 -o train_dataset2
```

PacBio reads simulation PacBio reads are simulated with `pbsim` with the following command. The model used is CLR, and the average sequence length is set to 3000bp with a minimum length of 400bp. The average accuracy is set to 0.78 with a standard deviation of 0.07. According to the authors [9], the reads are simulated with the CLR model, which has a 0.78 accuracy, 3.23% substitution rate, 10.53% insertion rate, and 3.98% deletion rate (obtained by aligning simulated reads to reference using LAST [11]). The coverage is also set to 20x.

```
$ pbsim [genome path] --prefix [output prefix] \
        --depth 20 --length-min 400 --length-mean 3000 \
        --accuracy-mean 0.78 --accuracy-sd 0.07 \
        --seed 522170 --model_qc [CLR model]
```

Nanopore reads simulation We used the NanoSim (v3.2.2) pre-trained metagenome model for bacteria community from `pre-trained_models/metagenome_ERR3152364_Even_v3.2.2.tar.gz` on the NanoSim GitHub site, which has a total error rate of 11.3% (3.9% mismatch, 3.2% insertion, and 4.2% deletion). We simulated 200,000, 400,000, and 200,000 reads for 50 known, 100 mixed, and 50 novel genomes accordingly, and only retained the reads deemed "alignable" by NanoSim (included in the output file `[prefix]_sample0_aligned_reads.fastq`). Due to the nature of NanoSim, the input abundance file represents the proportion of simulated bases from each included genome, instead of the actual abundance of represented species. The input abundance of each genome is set to the proportion of its genome size to the summed genome size of all included genomes so that all species abundances are equal. For example, a genome with 100,000 bp will be set to 10(%) if the summed genome size of all genomes is 1,000,000 bp. The DNA type of each input genome is set to circular. Finally, we used the following command for the Nanopore read simulation:

```
$ python3 NanoSim/src/simulator.py metagenome \
       -gl [genome path] -a [abundance path] \
       -dl [dnatype path] -c [pretrained model] \
       -o nanopore --seed [seed] --fastq -t 16
```

Genome accession numbers for reads simulation

1. **TIPP2 dataset 1 (33 genomes)**:

   GCA_002214465.1, GCA_002844335.1, GCA_002906575.1, GCA_002951815.1, GCA_003430825.1, GCA_006739055.1, GCA_006742785.1, GCA_011106835.1, GCA_011764545.1, GCA_012222825.1, GCA_013085545.1, GCA_013177635.1,

GCA_013177655.1, GCA_013201685.1, GCA_013201895.1, GCA_013267355.1, GCA_013283835.1, GCA_013347265.1, GCA_013391845.1, GCA_013394065.1, GCA_013409125.2, GCA_013415885.1, GCA_013466785.1, GCA_013488225.1, GCA_014042035.1, GCA_014076455.1, GCA_014076495.1, GCA_014189535.1, GCA_014217485.1, GCA_014218275.1, GCA_900324035.1, GCA_900631605.1, GCA_902702935.1

2. **TIPP2 dataset 2 (51 genomes)**:

   GCF_000020225.1, GCF_000265505.1, GCF_000238215.1, GCF_000183135.1, GCF_000190735.1, GCF_000190595.1, GCF_000020145.1, GCF_000069185.1, GCF_000092825.1, GCF_000023245.1, GCF_000023105.1, GCF_000063485.1, GCF_000020465.1, GCF_000218625.1, GCF_000484535.1, GCF_000020965.1, GCF_000021565.1, GCF_000183745.1, GCF_000191445.1, GCF_000192885.1, GCF_000018385.1, GCF_000018145.1, GCF_000009365.1, GCF_000020945.1, GCF_000022565.1, GCF_000266885.1, GCF_000195435.3, GCF_000017025.1, GCF_000163895.2, GCF_000147095.1, GCF_000024205.1, GCF_000025025.1, GCF_000018865.1, GCF_000017805.1, GCF_000021685.1, GCF_000024985.1, GCF_000092105.1, GCF_000024565.1, GCF_000025885.1, GCF_000279145.1, GCF_000010305.1, GCF_000441755.1, GCF_000253035.1, GCF_000298475.2, GCF_000237205.1, GCF_000092785.1, GCF_000286715.1, GCF_000018785.1, GCF_000021265.1, GCF_000284335.1, GCF_000235405.2

3. **Testing, 50 known genomes**:

   GCF_000020225.1, GCF_000265505.1, GCF_000238215.1, GCF_000183135.1, GCF_000190735.1, GCF_000190595.1, GCF_000020145.1, GCF_000092825.1, GCF_000023245.1, GCF_000023105.1, GCF_000063485.1, GCF_000020465.1, GCF_000218625.1, GCF_000484535.1, GCF_000020965.1, GCF_000021565.1, GCF_000183745.1, GCF_000191445.1, GCF_000192885.1, GCF_000018385.1, GCF_000018145.1, GCF_000009365.1, GCF_000020945.1, GCF_000022565.1, GCF_000266885.1, GCF_000195435.3, GCF_000017025.1, GCF_000163895.2, GCF_000147095.1, GCF_000024205.1, GCF_000025025.1, GCF_000018865.1, GCF_000017805.1, GCF_000021685.1, GCF_000024985.1, GCF_000092105.1, GCF_000024565.1, GCF_000025885.1, GCF_000279145.1, GCF_000010305.1, GCF_000441755.1, GCF_000253035.1, GCF_000298475.2, GCF_000237205.1, GCF_000092785.1, GCF_000286715.1, GCF_000018785.1, GCF_000021265.1, GCF_000284335.1, GCF_000235405.2

4. **Testing, 100 mixed genomes**:

   GCF_030758975.1, GCF_964019505.1, GCF_032268605.1, GCF_030490285.1, GCF_029625215.1, GCF_036670025.1, GCF_037948395.1, GCF_030285785.1, GCF_030408375.1, GCF_032594115.1, GCF_034554815.1, GCF_035930445.1, GCF_030296615.1, GCF_033344015.1, GCF_964020205.1, GCF_964020185.1, GCF_030719275.1, GCF_031885465.1, GCF_036352135.1, GCF_030296595.1, GCF_024346835.1, GCF_030419005.1, GCF_964019495.1, GCF_964019535.1, GCF_036204065.1, GCF_033126985.1, GCF_030389495.1, GCF_963556095.2, GCF_030540775.2, GCF_037076455.1, GCF_036250655.1, GCF_030687935.1, GCF_033095965.1, GCF_025232395.1, GCF_025736875.1, GCF_030296575.1, GCF_030409055.1, GCF_947241125.1, GCF_030177935.1, GCF_030296055.1, GCA_013409125.2, GCA_013200995.1, GCA_004295565.1, GCA_013201685.1, GCA_014217485.1, GCA_900000005.1, GCA_013177635.1, GCA_002813655.1, GCA_014236795.1, GCA_012225885.1, GCA_011455695.1, GCA_013391845.1, GCA_014042035.1, GCA_014069315.1, GCA_900631605.1, GCA_002156705.1, GCA_014048085.1, GCA_011462075.1, GCA_004799685.1, GCA_008326385.1,

GCA_000956175.1, GCA_002214465.1, GCA_002214385.1, GCA_002214485.1, GCA_013347265.1, GCA_003201835.2, GCA_013170725.1, GCA_009884315.1, GCA_013170785.1, GCA_013154935.1, GCA_011106835.1, GCA_014076455.1, GCA_013283835.1, GCA_013201895.1, GCA_013201665.1, GCA_013201935.1, GCA_009789175.1, GCA_004768745.1, GCA_013693755.1, GCA_013201825.1, GCA_008931705.1, GCA_002499975.2, GCA_001971705.1, GCA_002214505.1, GCA_002355655.1, GCA_002844335.1, GCA_013201725.1, GCA_000875775.1, GCA_003430825.1, GCA_013347305.1, GCA_002906575.1, GCA_009729015.1, GCA_002214565.1, GCA_006739055.1, GCA_012923785.1, GCA_003290265.1, GCA_012427845.1, GCA_013085545.1, GCA_003935895.2, GCA_900324035.1

5. **Testing, 50 novel genomes**:

   GCF_017357865.1, GCF_034262375.1, GCF_019880205.1, GCF_029101545.1, GCF_034258515.1, GCF_030316605.1, GCF_030252185.1, GCF_036492835.1, GCF_034479515.1, GCF_007833795.1, GCF_964019775.1, GCF_027563145.1, GCF_030438475.1, GCF_030407165.1, GCF_029023725.1, GCF_964019755.1, GCF_030294945.1, GCF_943169825.2, GCF_030408715.1, GCF_963675105.1, GCF_034479615.1, GCF_034371605.1, GCF_030295325.1, GCF_964019545.1, GCF_029101585.1, GCF_933509905.1, GCF_964019365.1, GCF_032397745.1, GCF_020865585.1, GCF_020524035.2, GCF_030622045.1, GCF_036320655.1, GCF_030296555.1, GCF_030408455.1, GCF_027923555.1, GCF_034359465.1, GCF_030294405.1, GCF_033472595.1, GCF_031312535.1, GCF_008726475.3, GCF_030406165.1, GCF_036178885.1, GCF_018128945.1, GCF_030845235.1, GCF_005517195.1, GCF_030758955.1, GCF_030550815.1, GCF_964020195.1, GCF_030408755.1, GCF_026250525.1

CAMI-II dataset We used replicate 1 of the CAMI-II Marine dataset with short (Illumina) and long (PacBio) reads. The Marine dataset originally contains 777 species, but only 476 were simulated reads with any abundance in replicate 1. The dataset can be downloaded from `https://frl.publisso.de/data/frl:6425521/marine/`.

D Additional Information for Normalized Hellinger Distance

D.1 Normalized Hellinger distance

Remember that regular Hellinger distance is given by:

$$H_l = \frac{\sqrt{\sum_{x \in C_l} (\sqrt{T_x} - \sqrt{E_x})^2}}{\sqrt{2}},$$

where T_x is the true abundance and E_x the estimated abundance of a clade x, for each x in the set of clades C_l on a taxonomic level l. Reads that are unclassified at a certain level are not counted for the Hellinger distance calculation.

Theorem D.1 (Theoretically Worst Hellinger Distance)**.** *If an abundance profiling method classifies n reads in total, and n_l reads are assigned with taxonomic labels at the taxonomic level l, then the theoretically worst Hellinger distance that the method can have at taxonomic level l is $H_l' = \frac{\sqrt{1+\frac{n_l}{n}}}{\sqrt{2}}$.*

Proof. In the worst case, an estimated profile is disjoint from the true profile at taxonomic level l. More formally this means that for any clade $x \in C_l$, where C_l is the union of labels in the estimated and true profiles, $T_x = 0$ or $E_x = 0$, exclusively. This also means $(\sqrt{T_x} - \sqrt{E_x})^2$ is either T_x or E_x, depending on if clade x is defined in the estimated or the true profile.

While the true profile sums to 1 by definition (i.e., taxonomic labels are known for reads), only $\frac{n_l}{n}$ of the estimated profile counts toward the Hellinger distance computation at level l. Then, we can rewrite the Hellinger distance computation as:

$$\begin{aligned} H_l' &= \frac{\sqrt{\sum_{x \in C_l} (\sqrt{T_x} - \sqrt{E_x})^2}}{\sqrt{2}} \\ &= \frac{\sqrt{\sum_{x \in C_l} T_x + \sum_{x \in C_l} E_x}}{\sqrt{2}} \\ &= \frac{\sqrt{1 + \frac{n_l}{n}}}{\sqrt{2}} \end{aligned}$$

□

By Theorem D.1, the Hellinger distance H_l of an estimated profile is bounded by $0 \leq H_l \leq H_l'$ at a taxonomic level l, and $H_l' \leq 1$. Only when all classified reads are assigned taxonomic labels at level l ($n_l = n$), the range is from 0 to 1. Therefore, when H_l is used to measure a method for abundance profiling accuracy, it would be biased if the method fails to classify all reads at level l.

A simple alternative is to measure profiling accuracy by $H_l^* = H_l / H_l'$, normalizing the measurement range to $[0, 1]$ and being independent of the number of reads classified at a taxonomic level for each method (n_l). We refer to this measurement as **normalized Hellinger distance**.

$$H_l^* = \frac{H_l}{H_l'} = \frac{\sqrt{\sum_{x \in C_l} (\sqrt{T_x} - \sqrt{E_x})^2}}{\sqrt{1 + \frac{n_l}{n}}}$$

D.2 Example for biases with Hellinger distance

Regular Hellinger distance is biased when the profile has a high fraction of unclassified reads. For example, let n be the total number of reads classified. At the species level, let a method A in total estimate 5 types of species $[s_1, s_2, s_3, s_4, s_5]$, where $s_i = 0.1, i = 1, 2, 3, 4, 5$. Then, the proportion of classified reads at taxonomic level l for method A is $n_{l,A} = 0.5$ and $n_{l,A}/n = 0.5$, with the other 0.5 as "unclassified/unspecified". In calculating Hellinger distance $H_{l,A}$ between the profile of method A to the reference profile at level l, only the 0.5 that is classified/specified is used. Hence, the theoretically worst Hellinger distance $H'_{l,A}$ that method A can have is to have a disjoint profile to the reference, meaning that $H'_{l,A} = \sqrt{1 + 0.5}/\sqrt{2} = 0.866$. In other words, given a method's estimated profile, H'_l is the actual upper bound for H_l.

In the most extreme case when a method B fails to produce *any classified groups* and has 1.0 as unclassified, then $H'_{l,B} = H_{l,B} = \sqrt{1 + 0}/\sqrt{2} = 0.707$. Let's assume that the reference profile looks like $[s_{100} = 0.7, s_{150} = 0.3]$. Another method C may make some meaningful estimation, such as $[s_1 = 0.1, s_2 = 0.1, s_3 = 0.1, s_4 = 0.1, s_{100} = 0.05]$, as it gets only one group overlapping with the reference. If we compute the Hellinger distance for method C, we will get:

$$\begin{aligned} H_{l,C} &= \frac{\sqrt{\sum_{x \in C_l} (\sqrt{T_x} - \sqrt{E_x})^2}}{\sqrt{2}} \\ &= \frac{\sqrt{0.3 + 0.1 \cdot 4 + (\sqrt{0.7} - \sqrt{0.05})^2}}{\sqrt{2}} \\ &= 0.733 \end{aligned}$$

We can see that although method B does not give any estimation, it still gets a lower Hellinger distance than method C, which indicates a bias of Hellinger distance that favors making no estimation.

On the other hand, if we compare the normalized Hellinger distances of the two methods, we have:

$$\begin{aligned} H_{l,B}/H'_{l,B} &= 0.707/0.707 = 1 \\ H_{l,C}/H'_{l,C} &= 0.733/(\sqrt{1.45}/\sqrt{2}) = 0.861 \end{aligned}$$

where we have method C having a lower error, which makes more sense.

E Additional Results for Experiment 1: Designing TIPP3

E.1 Adding reads to marker gene alignments

We obtained UPP and WITCH alignments of reads binned to marker genes RplO, RpsK, and RpsL. WITCH was run with different parameter settings, varying the number of hidden Markov models (HMMs) for aligning each read and the decomposition subset sizes (i.e., how many sequences to include in a subset to build an HMM on). Then, BSCAMPP [12] was used to obtain placement and taxonomic identification of each read in each marker gene taxonomy.

Based on overall taxonomic identification accuracy, we selected WITCH with one hidden Markov model subset and subset size ranging from 10 to 1000 sequences as our query read alignment method. Full results for the alignment benchmarks can be found below.

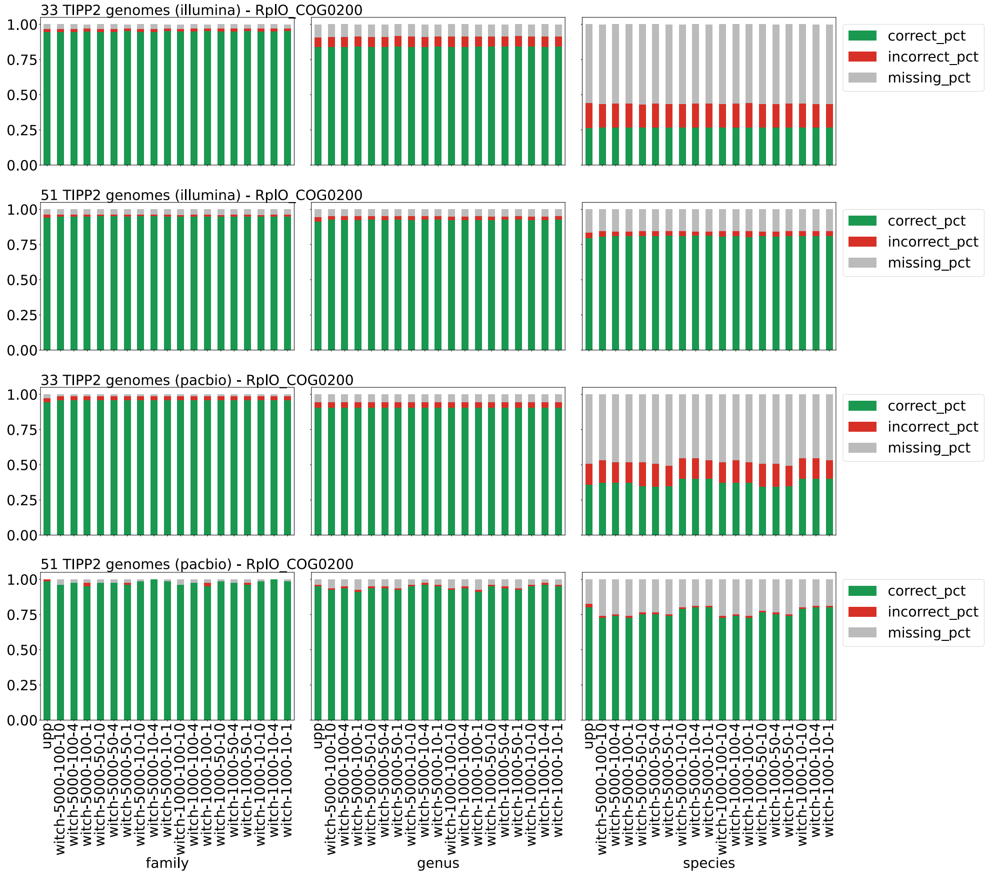

Fig A. Experiment 1: Taxonomic identification accuracy of UPP and different WITCH alignment variants on marker gene RplO_COG0200, for Illumina and PacBio reads from two training datasets with 33 (mixed) and 51 (known) genomes (Family, Genus, and Species levels). The top two panels are for Illumina reads, and the bottom two are for PacBio reads. Taxonomic identification is done by performing query placements with BSCAMPP with a subtree size of 1000 and a support value of 95%. WITCH variants are referred to as **witch-Z-A-k**, for which Z denotes the upper bound for subset size, A the lower bound, and k the number of subsets to align a query read. "correct_pct" denotes the fraction of correctly identified reads (at a taxonomic level), "incorrect_pct" denotes the fraction of incorrectly identified reads, and "missing_pct" denotes the fraction of not-identified reads. The three fractions sum to 1 for each bar.

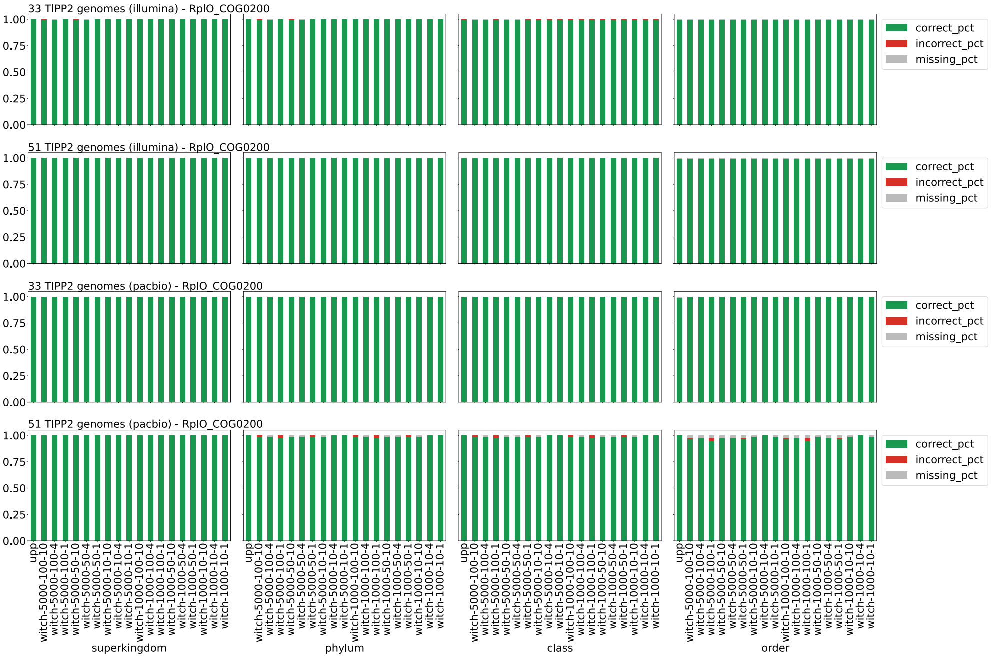

Fig B. Experiment 1: Taxonomic identification accuracy of UPP and different WITCH alignment variants on marker gene RplO_COG0200, for Illumina and PacBio reads from two training datasets with 33 (mixed) and 51 (known) genomes (Superkingdom, Phylum, Class, and Order levels). The top two panels are for Illumina reads, and the bottom two are for PacBio reads. Taxonomic identification is done by performing query placements with BSCAMPP with a subtree size of 1000 and a support value of 95%. WITCH variants are referred to as **witch-Z-A-k**, for which Z denotes the upper bound for subset size, A the lower bound, and k the number of subsets to align a query read. "correct_pct" denotes the fraction of correctly identified reads (at a taxonomic level), "incorrect_pct" denotes the fraction of incorrectly identified reads, and "missing_pct" denotes the fraction of not-identified reads. The three fractions sum to 1 for each bar.

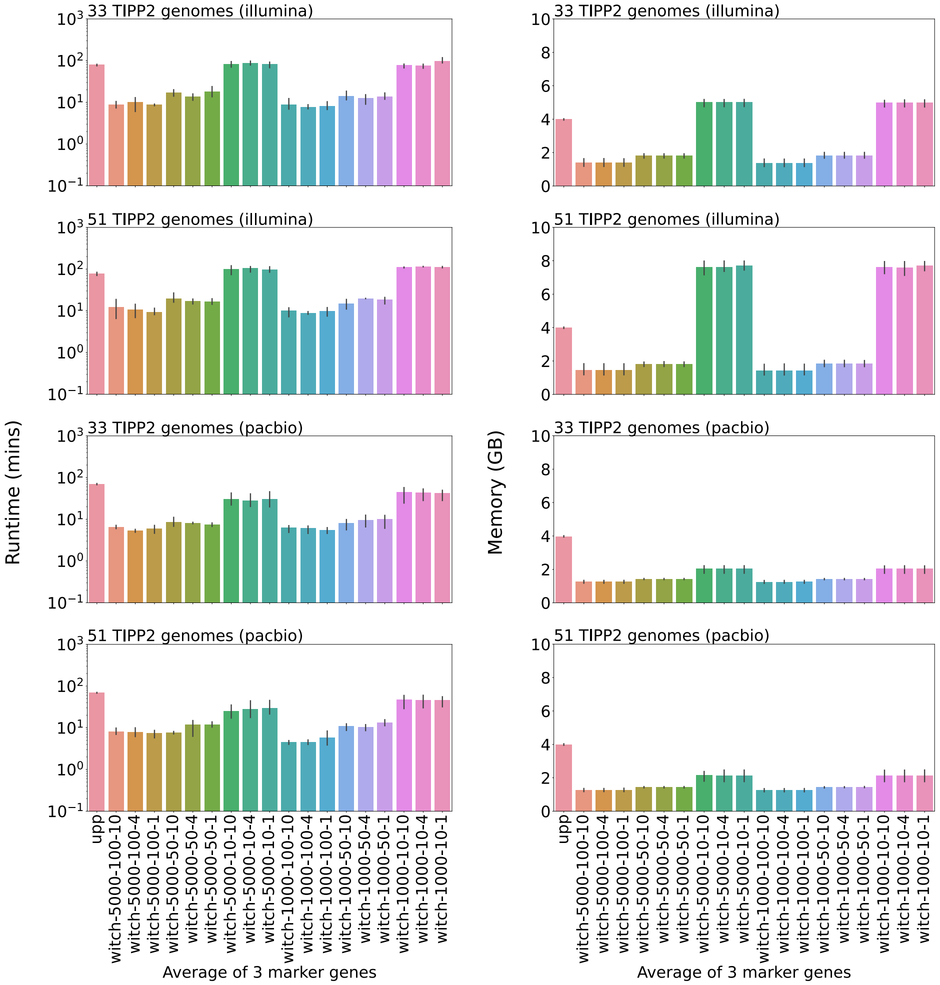

(a) Runtime (mins, log-scale). **(b)** Memory (GBs).

Fig C. Experiment 1: Runtime usage in minutes (left, log-scale) and memory usage in GBs (right) of identifying Illumina and PacBio reads from two training datasets with 33 (mixed) and 51 (known) genomes, by UPP and different WITCH variants. Results are averaged over three marker genes (RplO, RpsK, and RpsL). Runtime for each variant includes read alignment and placement phases.

E.2 Placing reads into marker gene taxonomies

After selecting the read alignment method, we explored different phylogenetic placement methods that place aligned reads into a target tree (i.e., the taxonomy of each marker gene), including SCAMPP [13], BSCAMPP [12], and pplacer with the `taxtastic` package (referred to as pplacer-taxtastic) [4, 14]. Both SCAMPP and BSCAMPP are divide-and-conquer methods that decompose the input tree into smaller subtrees and select suitable subtrees for query placements, using pplacer (SCAMPP) or EPA-ng [15] (BSCAMPP). We varied subtree sizes ranging from 1000 to 5000 leaves for SCAMPP and BSCAMPP, and support values ranging from 50% to 99% for all three methods (see main text for the definition of a support value). We then examined the taxonomic identification and abundance profiling accuracy of each method variant.

Overall, pplacer-taxtastic with a support value of 90% provides the best accuracy, while BSCAMPP with a subtree of size 1000 and a support value of 95% gives good accuracy and the fastest runtime. Hence, we selected pplacer with the `taxtastic` package (90% support value) as the read placement method for TIPP3. Full results for the placement benchmarks can be found below.

E.2.1 BSCAMPP

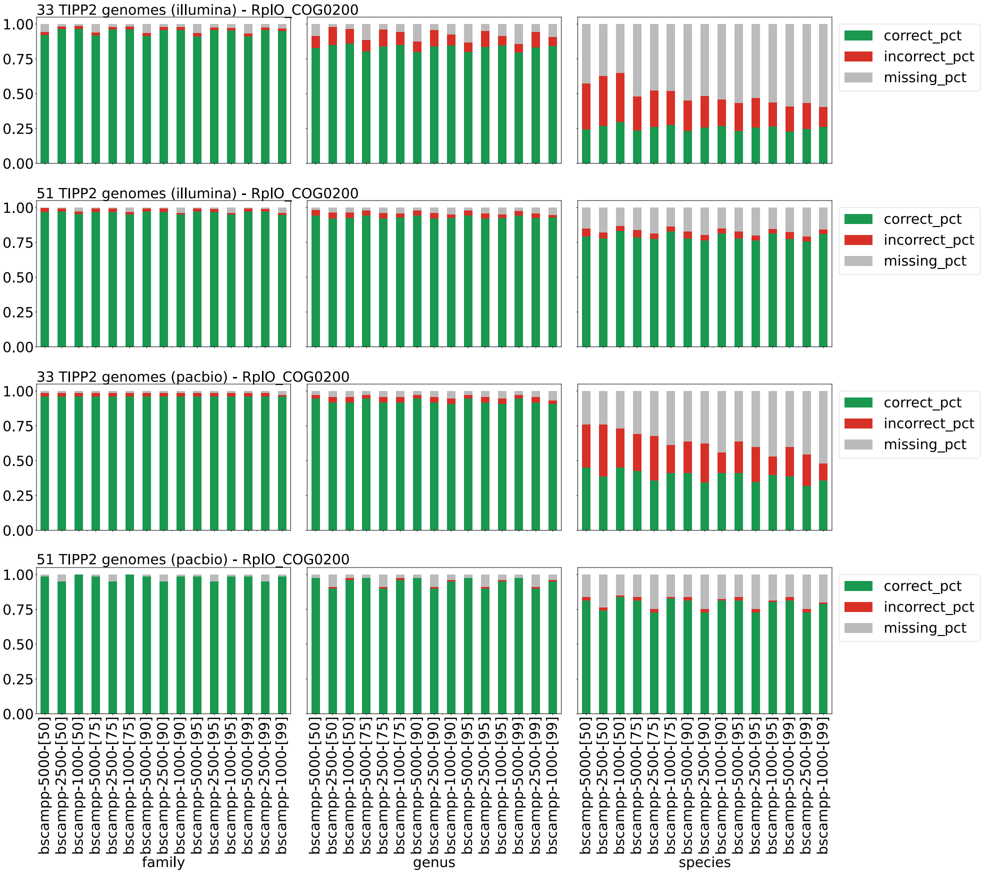

Fig D. Experiment 1: Taxonomic identification accuracy of different BSCAMPP variants for query placement in TIPP3 on marker gene RplO_COG0200, for Illumina and PacBio reads from two training datasets with 33 (mixed) and 51 (known) genomes (Family, Genus, and Species levels). Query reads are aligned with WITCH. BSCAMPP variants are named **bscampp-X-[Y]**, where X is the subtree size ($X = \{1000, 2500, 5000\}$) and Y is the support value ($Y = \{50, 75, 90, 95, 99\}$). "correct_pct" denotes the fraction of correctly identified reads (at a taxonomic level), "incorrect_pct" denotes the fraction of incorrectly identified reads, and "missing_pct" denotes the fraction of not-identified reads. The three fractions sum to 1 for each bar.

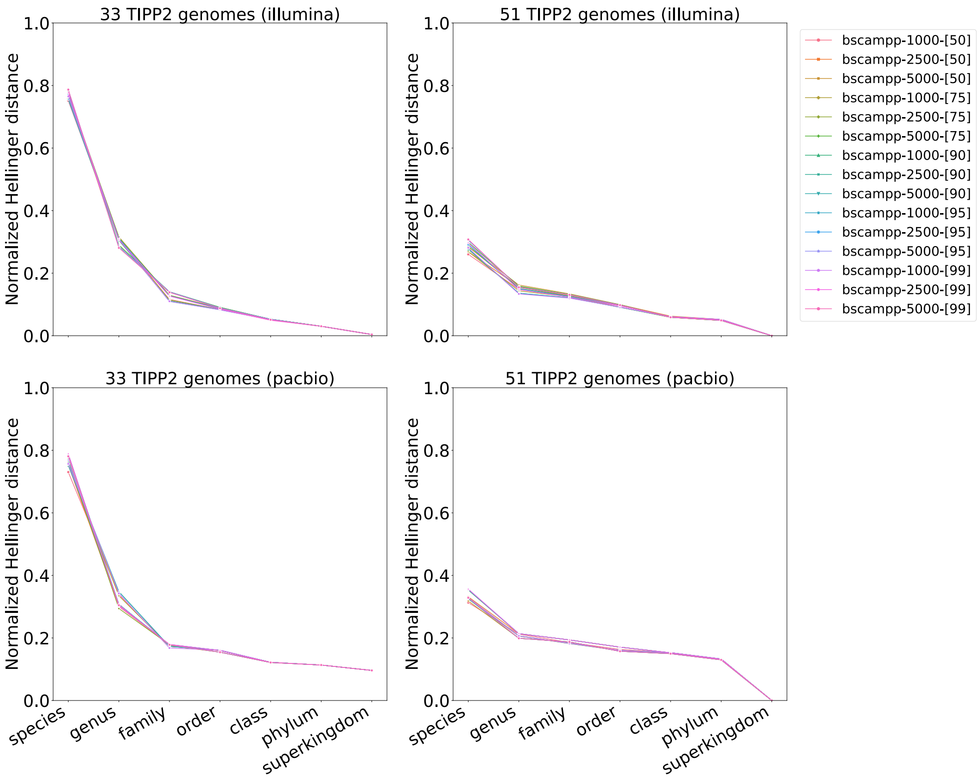

Fig E. Experiment 1: Abundance profile of different BSCAMPP variants for query placement in TIPP3, for Illumina and PacBio reads from two training datasets with 33 (mixed) and 51 (known) genomes. Query reads are aligned with WITCH. The abundance profile is computed as the normalized Hellinger distance between the estimated and reference profiles using three marker genes, RplO, RpsK, and RpsL.

E.2.2 SCAMPP

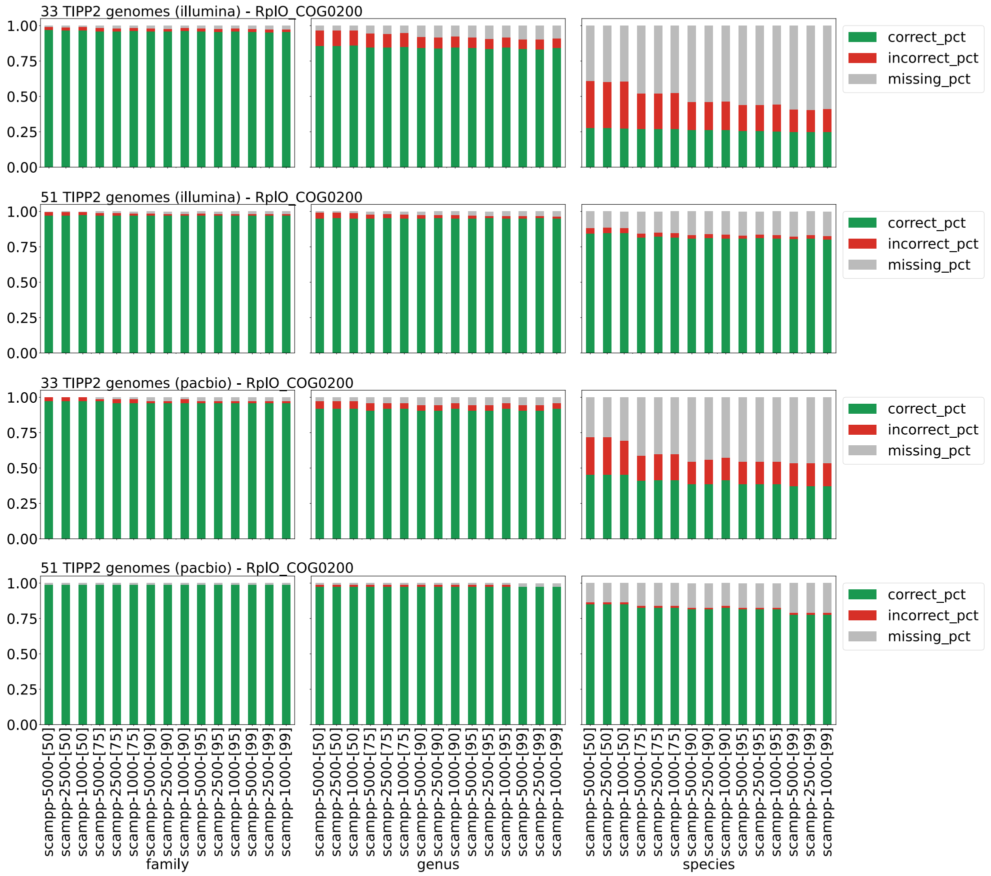

Fig F. Experiment 1: Taxonomic identification accuracy of different SCAMPP variants for query placement in TIPP3 on marker gene RplO_COG0200, for Illumina and PacBio reads from two training datasets with 33 (mixed) and 51 (known) genomes(Family, Genus, and Species levels). Query reads are aligned with WITCH. SCAMPP variants are named **scampp-X-[Y]**, where X is the subtree size ($X = \{1000, 2500, 5000\}$) and Y is the support value ($Y = \{50, 75, 90, 95, 99\}$). "correct_pct" denotes the fraction of correctly identified reads (at a taxonomic level), "incorrect_pct" denotes the fraction of incorrectly identified reads, and "missing_pct" denotes the fraction of not-identified reads. The three fractions sum to 1 for each bar.

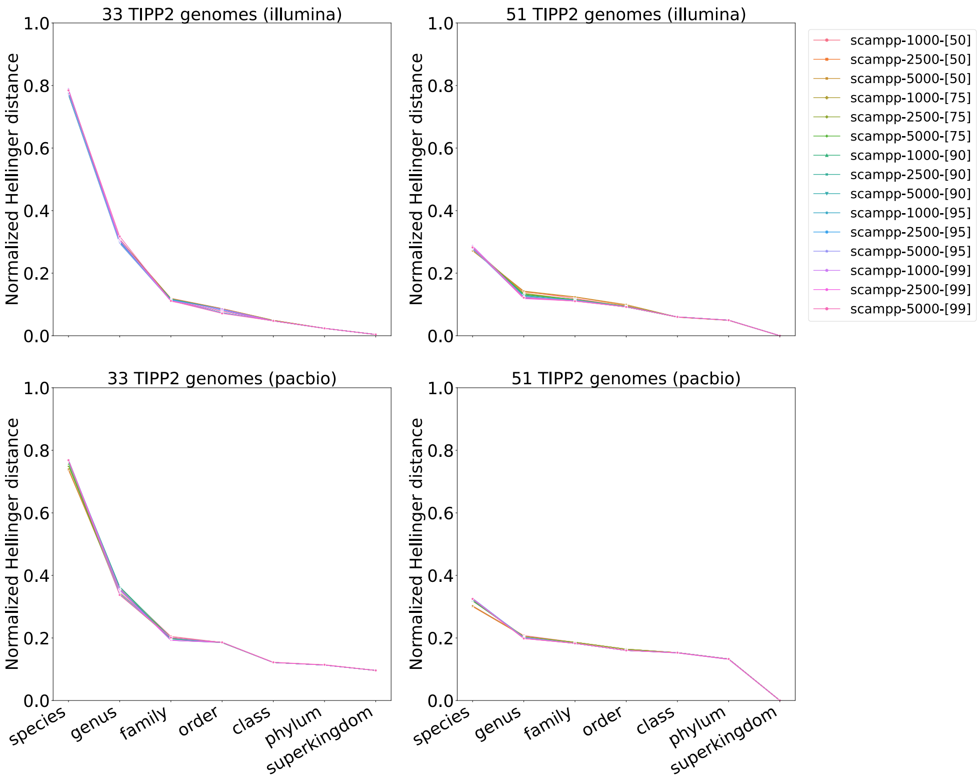

Fig G. Experiment 1: Abundance profile of different SCAMPP variants for query placement in TIPP3, for Illumina and PacBio reads from two training datasets with 33 (mixed) and 51 (known) genomes. Query reads are aligned with WITCH. The abundance profile is computed as the normalized Hellinger distance between the estimated and reference profiles using three marker genes, RplO, RpsK, and RpsL.

E.2.3 pplacer-taxtastic

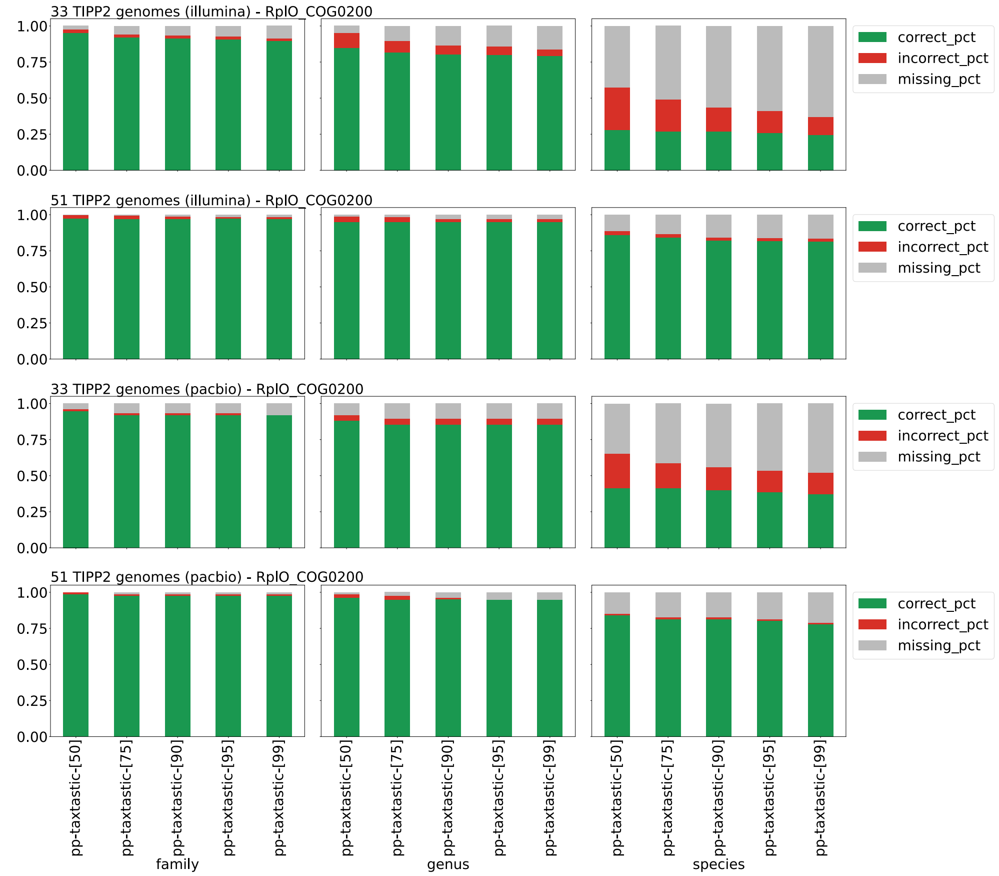

Fig H. Experiment 1: Taxonomic identification accuracy of different pplacer-taxtastic variants for query placement in TIPP3 on marker gene RplO_COG0200, for Illumina and PacBio reads from two training datasets with 33 (mixed) and 51 (known) genomes (Family, Genus, and Species levels). Query reads are aligned with WITCH. pplacer-taxtastic variants are named **pp-taxtastic-[Y]**, where Y is the support value ($Y = \{50, 75, 90, 95, 99\}$). "correct_pct" denotes the fraction of correctly identified reads (at a taxonomic level), "incorrect_pct" denotes the fraction of incorrectly identified reads, and "missing_pct" denotes the fraction of not-identified reads. The three fractions sum to 1 for each bar.

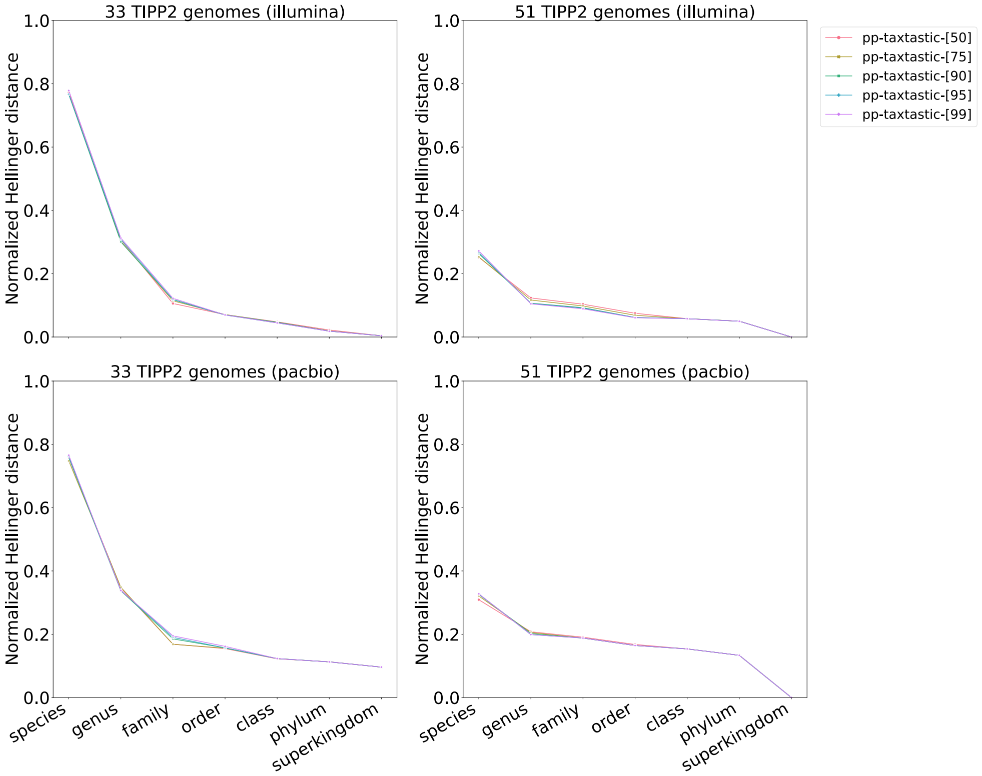

Fig I. Experiment 1: Abundance profile of different pplacer-taxtastic variants for query placement in TIPP3, for Illumina and PacBio reads from two training datasets with 33 (mixed) and 51 (known) genomes. Query reads are aligned with WITCH. The abundance profile is computed as the normalized Hellinger distance between the estimated and reference profiles using three marker genes, RplO, RpsK, and RpsL.

E.2.4 Comparison of the three placement methods

342

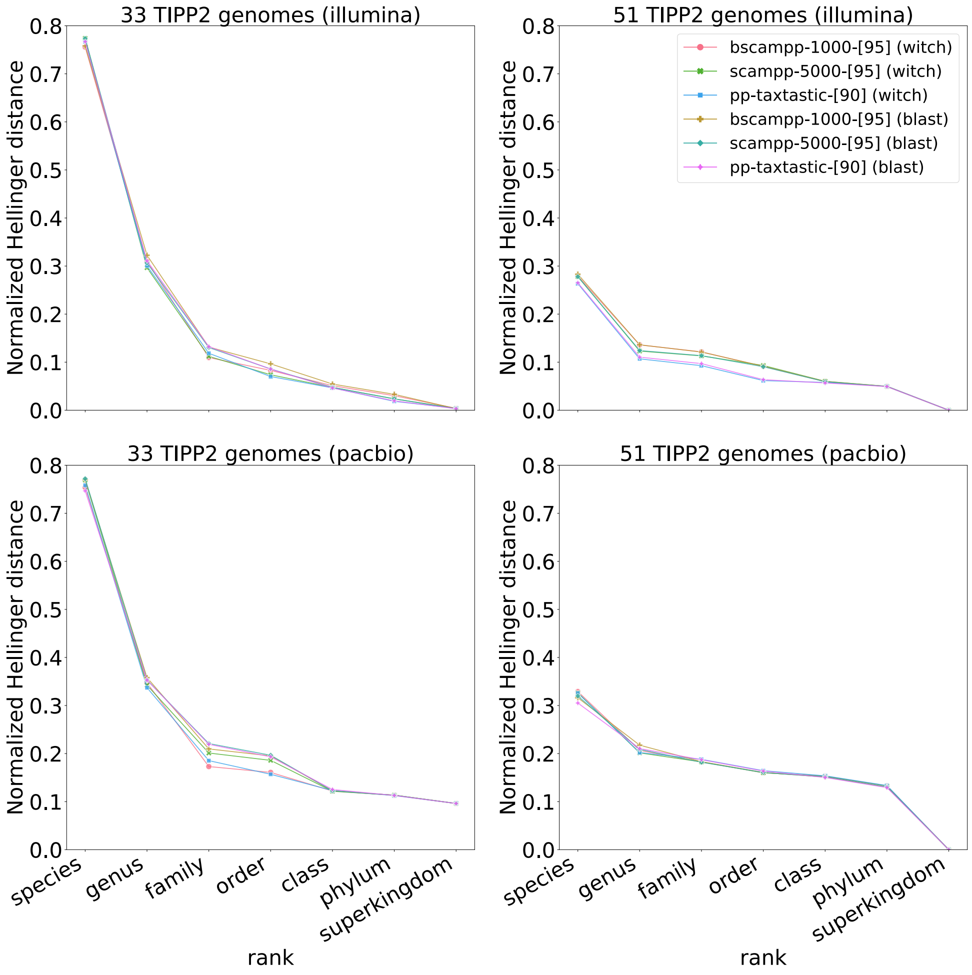

Fig J. Experiment 1: Abundance profile of the best variants of BSCAMPP, SCAMPP, and pplacer-taxtastic for Illumina and PacBio reads from the Training datasets with 33 (mixed) and 51 (known) genomes. Query reads are aligned with either WITCH or BLAST (denoted after the name of each method). The abundance profile is computed as the normalized Hellinger distance between the estimated and reference profiles using three marker genes, RplO, RpsK, and RpsL.

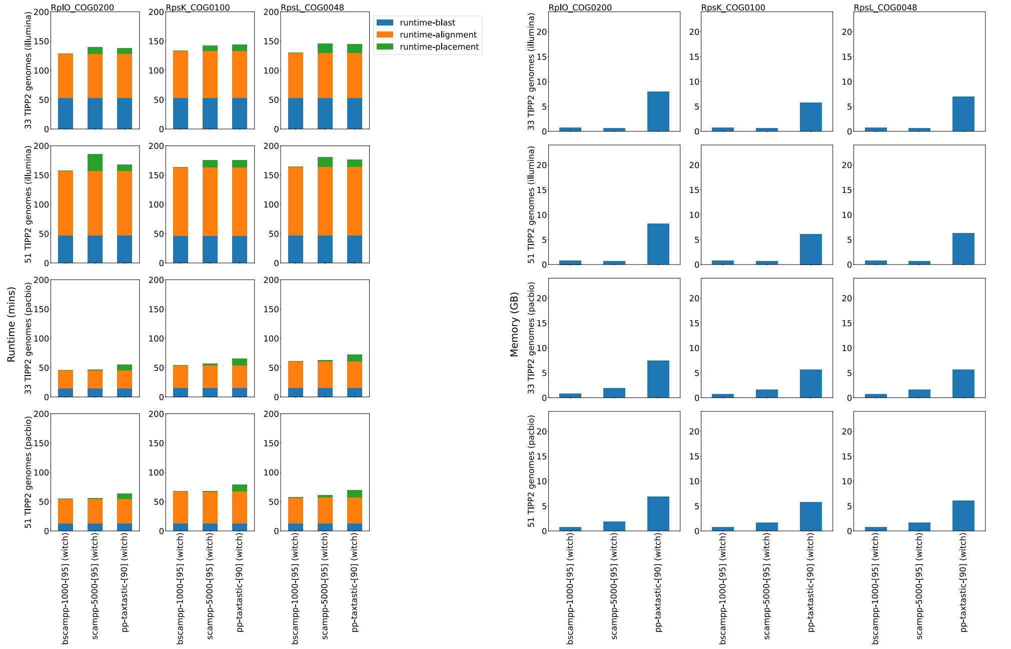

(a) Runtime (mins). **(b)** Memory (GBs).

Fig K. Experiment 1: Runtime usage in minutes (left) and memory usage in GBs (right), using WITCH alignment, of the best variants of BSCAMPP, SCAMPP, and pplacer-taxtastic identifying Illumina and PacBio reads from the training datasets (33 mixed and 51 known genomes) assigned to three marker genes, RplO, RpsK, and RpsL. Runtime for blast, alignment (by WITCH), and placement are shown. "runtime-alignment" denotes the time to obtain the read alignment by WITCH assigned to each marker gene. "runtime-placement" denotes the total time used to obtain placements of query reads assigned to each marker gene.

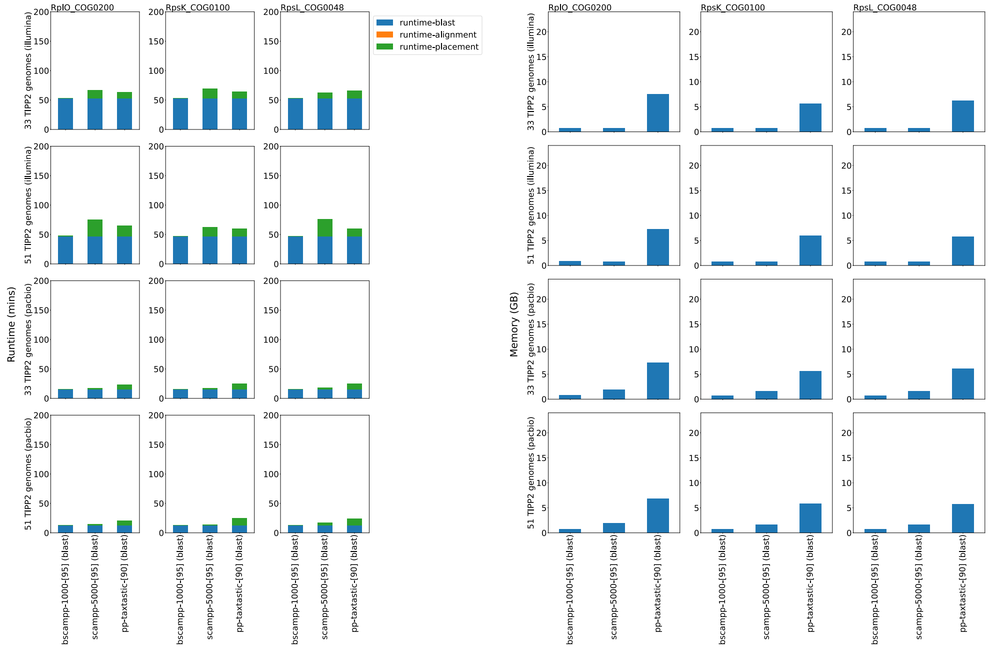

(a) Runtime (mins). **(b)** Memory (GBs).

Fig L. Experiment 1: Runtime usage in minutes (left) and memory usage in GBs (right), using BLAST alignment, of the best variants of BSCAMPP, SCAMPP, and pplacer-taxtastic identifying Illumina and PacBio reads from the training datasets (33 mixed and 51 known genomes) assigned to three marker genes, RplO, RpsK, and RpsL. Runtime for blast, alignment (by BLAST), and placement are shown. "runtime-alignment" denotes the time to post-process the BLAST output to obtain best-scored pairwise alignments (to reference genomes) of binned query reads. "runtime-placement" denotes the total time used to obtain placements of query reads assigned to each marker gene.

E.3 Marker gene selection

So far, we have only looked at three marker genes (RplO, RpsK, and RpsL) for abundance profiling. Here, we examined the accuracy of TIPP3 on each of the 40 marker genes when profiling Illumina reads from the TIPP2 dataset with 51 genomes. We then sorted the marker genes by their averaged normalized Hellinger distance across all taxonomic levels (Figure M). Full results can be found in this sub-section.

We observed a wide gap between the best and the worst marker genes in terms of their profiling accuracy, particularly on the genus level and up. When using a subset of marker genes for aggregated abundance profiling, we found that excluding either the largest two marker genes (FtsY and RpoB, ranked by marker gene alignment sizes) or the bottom 20 marker genes generally resulted in higher profiling accuracy compared to using all 40 marker genes or just three marker genes (Figure N). Hence, we removed FtsY and RpoB genes from the TIPP3 reference package and used the remaining 38 marker genes for the following experiments.

E.3.1 Abundance profile on each marker gene

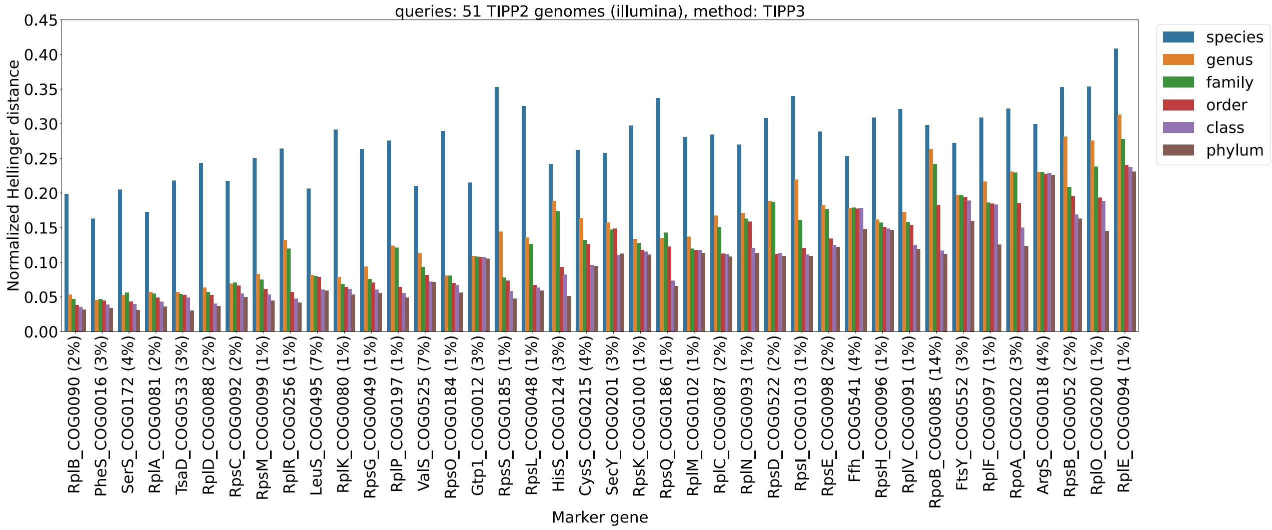

Fig M. Experiment 1: Individual marker gene abundance profile accuracy for TIPP3 on each taxonomic level. The query reads used are Illumina reads and simulated from the TIPP2 dataset with 51 genomes. The x-axis denotes different marker genes and taxonomic levels (excluding superkingdom). The marker genes are sorted from left to right in order of average ranking of normalized Hellinger distance (i.e., the lower average normalized Hellinger distance a marker gene has, the closer it is to left on the x-axis). The “%” sign for each marker gene denotes the percentage of query reads assigned to that marker gene.

E.3.2 Abundance profile using different sets of marker genes

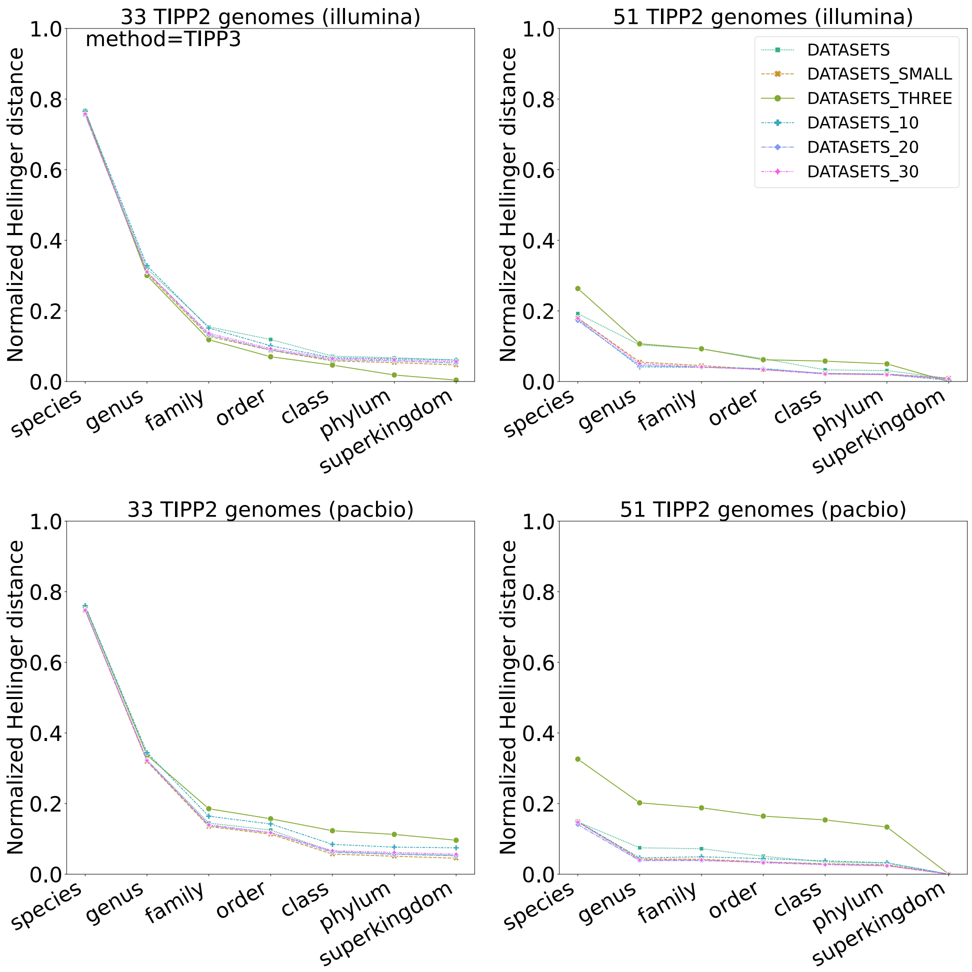

Fig N. Experiment 1: Abundance profiles of TIPP3 using different sets of marker genes. DATASETS denotes using all marker genes, DATASETS_SMALL for using all except RpoB and FtsY, DATASETS_THREE for using only RplO, RpsK, and RpsL, DATASETS_10 for using the top 10 marker genes denoted in Figure M, DATASETS_20 for using the top 20, and DATASETS_30 for using the top 30 marker genes. Reads are either Illumina or PacBio simulated from the training datasets (33 mixed and 51 known genomes).

F Additional Results for Experiment 2: Restricting methods to filtered reads

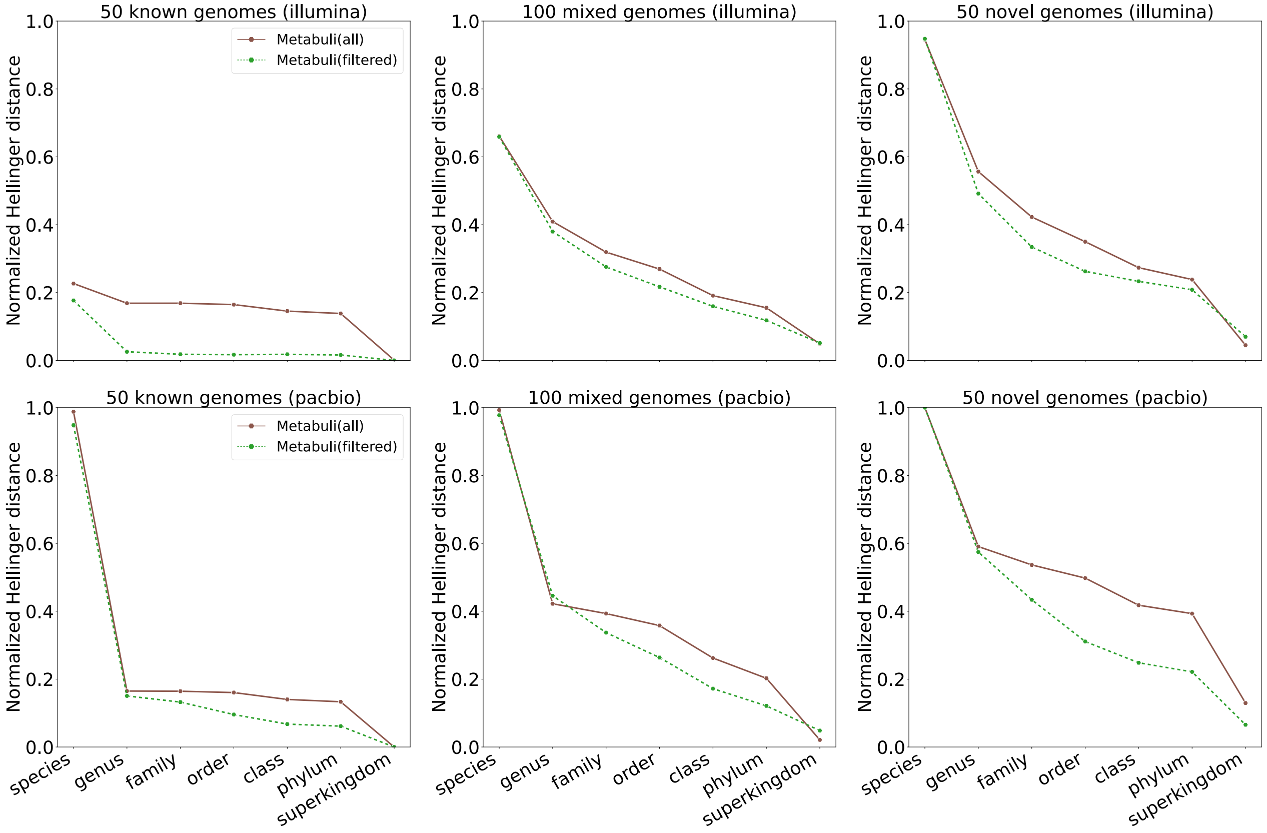

Fig O. Experiment 3: Abundance profiling accuracy by normalized Hellinger distance (lower means more accurate) of two ways of running Metabuli on three mock microbial communities (50 known, 100 mixed, and 50 novel genomes). Dashed lines correspond to using filtered reads, and solid lines correspond to using all (unfiltered) reads.

G Additional Results for Experiment 3: Evaluation of TIPP3 for abundance profiling

G.1 Abundance profile comparison for all methods

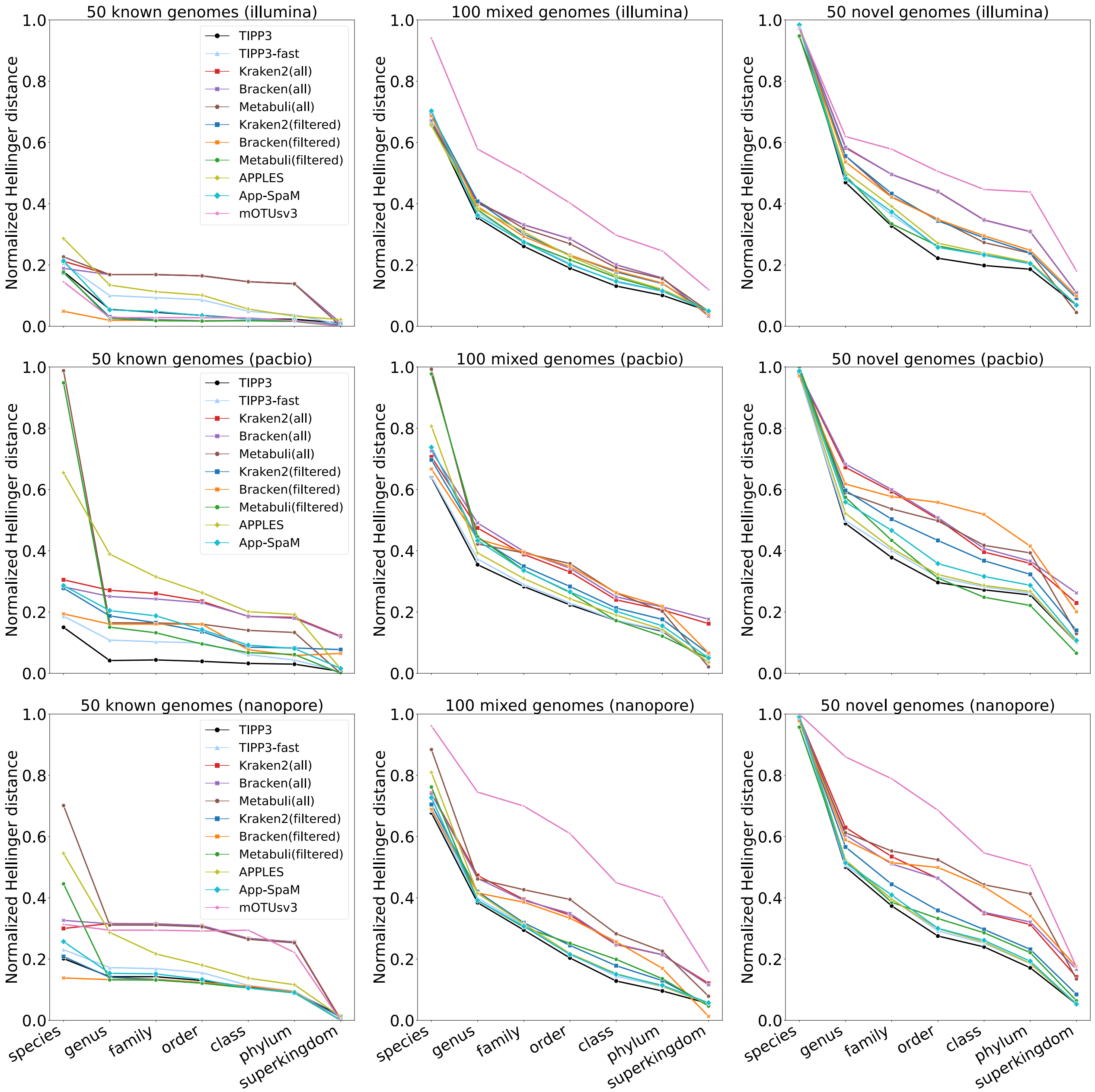

Fig P. Experiment 3: Abundance profiling accuracy by normalized Hellinger distance (lower means more accurate) of all methods/pipelines for Illumina, PacBio, and Nanopore reads from three mock microbial communities (50 known, 100 mixed, and 50 novel genomes). For PacBio read datasets, mOTUsv3 did not produce any classification and thus is not shown.

G.2 Detailed evaluation on species and genus abundances

Genus- and Species-specific abundance estimation errors are shown. Species-specific errors are not shown for 50 novel genomes, since no methods should be able to produce a non-zero profile for the included species by definition.

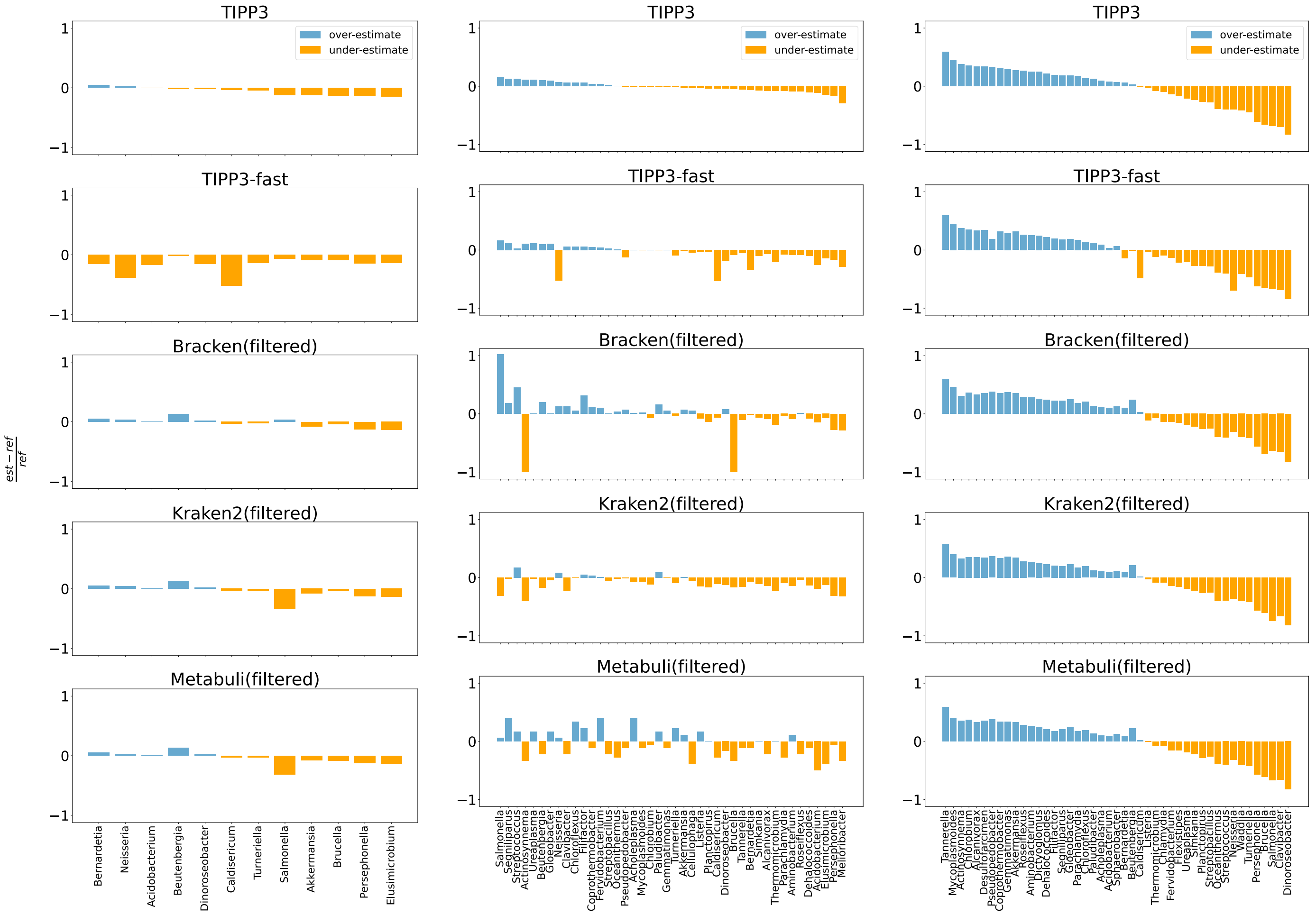

(a) Illumina reads. **(b)** PacBio reads. **(c)** Nanopore reads.

Fig Q. Experiment 3: Genus-specific abundance estimation error for Illumina (left), PacBio (middle), and Nanopore (right) reads of 50 known genomes of TIPP3, TIPP3-fast, Bracken(filtered), Kraken2(filtered), and Metabuli(filtered). The estimation error of a taxon is computed as the fractional difference between its estimated and reference compositions, shown on the y-axis. Taxa are sorted by the estimation errors by TIPP3 in ascending order. A taxon group is shown if and only if it is present in the reference, and any of the methods has an estimation error greater than 10% in magnitude

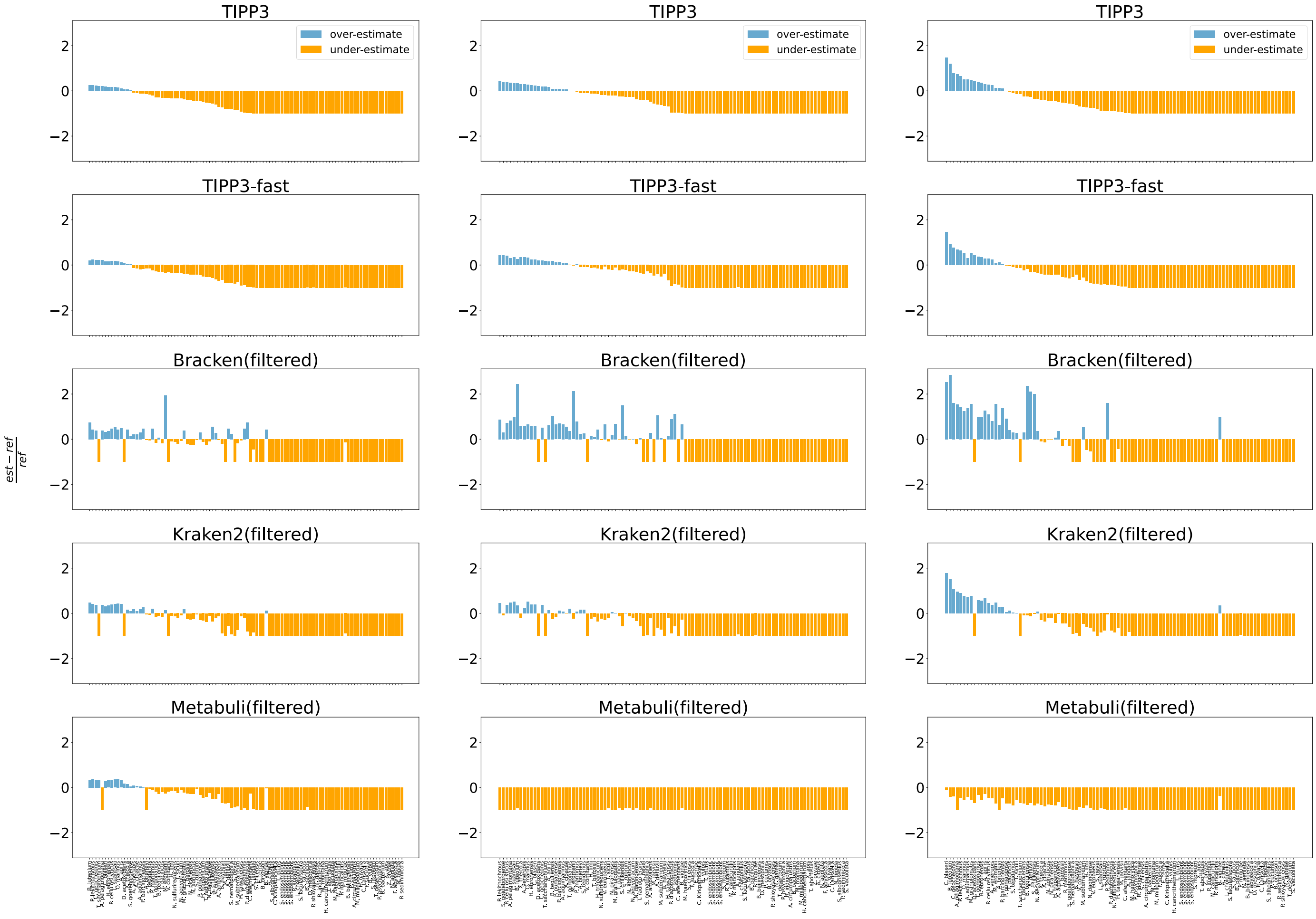

(a) Illumina reads. **(b)** PacBio reads. **(c)** Nanopore reads.

Fig R. Experiment 3: Species-specific abundance estimation error for Illumina (left), PacBio (middle), and Nanopore (right) reads of 100 mixed genomes of TIPP3, TIPP3-fast, Bracken(filtered), Kraken2(filtered), and Metabuli(filtered). The estimation error of a taxon is computed as the fractional difference between its estimated and reference compositions, shown on the y-axis. Taxa are sorted by the estimation errors by TIPP3 in ascending order. A taxon group is shown if and only if it is present in the reference, and any of the methods has an estimation error greater than 10% in magnitude.

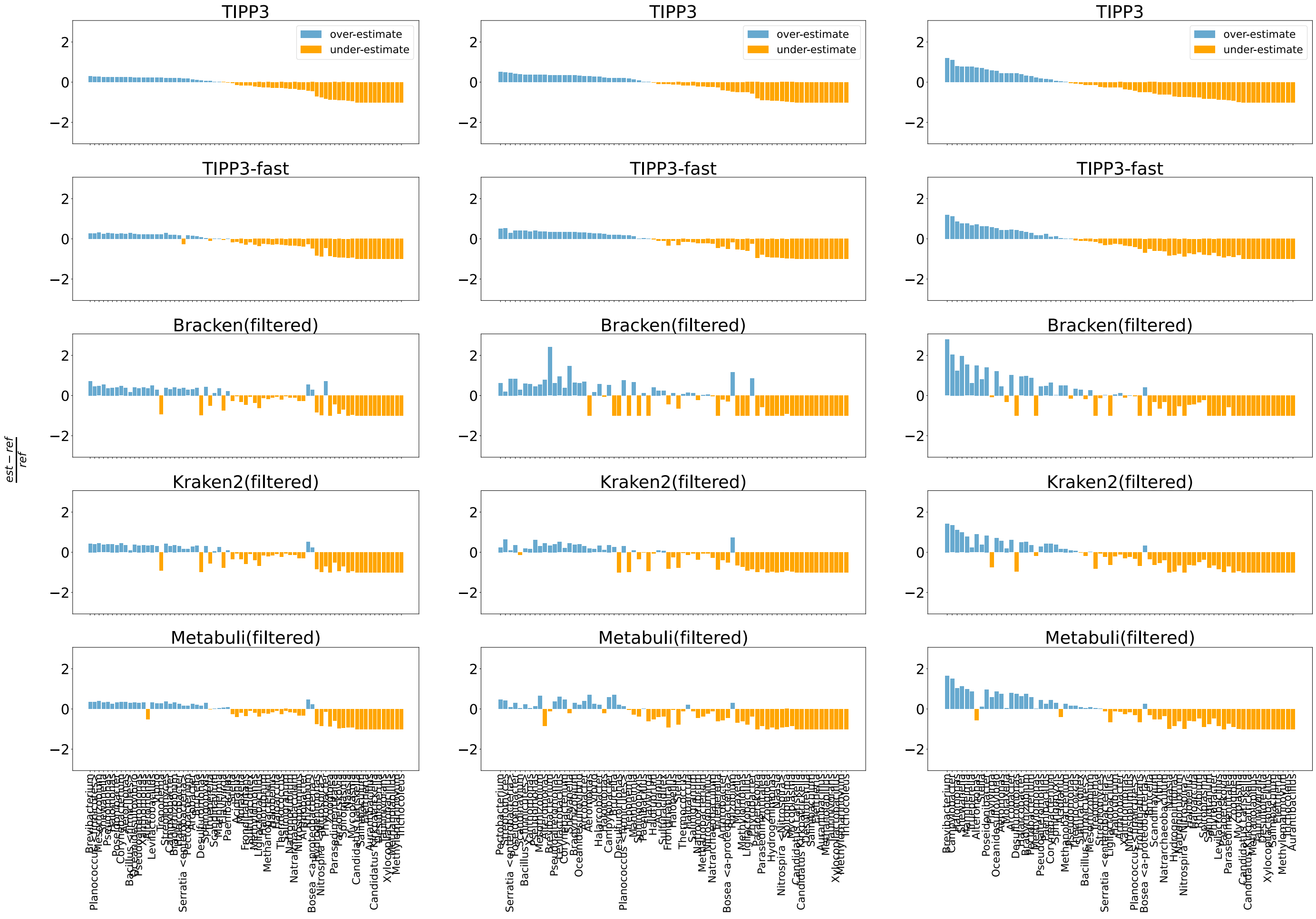

(a) Illumina reads. **(b)** PacBio reads. **(c)** Nanopore reads.

Fig S. Experiment 3: Genus-specific abundance estimation error for Illumina (left), PacBio (middle), and Nanopore (right) reads of 100 mixed genomes of TIPP3, TIPP3-fast, Bracken(filtered), Kraken2(filtered), and Metabuli(filtered). The estimation error of a taxon is computed as the fractional difference between its estimated and reference compositions, shown on the y-axis. Taxa are sorted by the estimation errors by TIPP3 in ascending order. A taxon group is shown if and only if it is present in the reference, and any of the methods has an estimation error greater than 10% in magnitude.

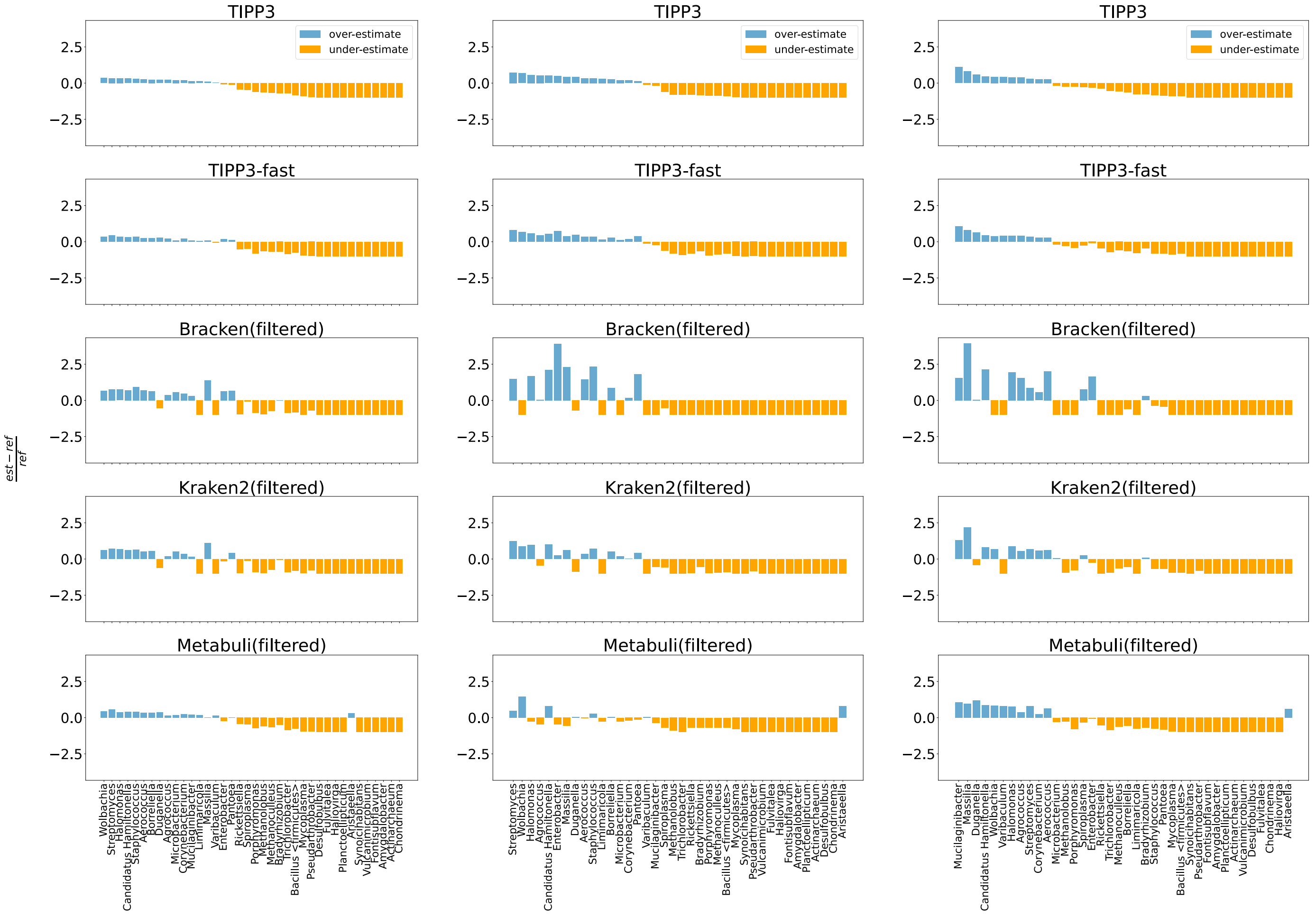

(a) Illumina reads. **(b)** PacBio reads. **(c)** Nanopore reads.

Fig T. Experiment 3: Genus-specific abundance estimation error for Illumina (left), PacBio (middle), and Nanopore (right) reads of 50 novel genomes of TIPP3, TIPP3-fast, Bracken(filtered), Kraken2(filtered), and Metabuli(filtered). The estimation error of a taxon is computed as the fractional difference between its estimated and reference compositions, shown on the y-axis. Taxa are sorted by the estimation errors by TIPP3 in ascending order. A taxon group is shown if and only if it is present in the reference, and any of the methods has an estimation error greater than 10% in magnitude..

G.3 Runtime and memory usage

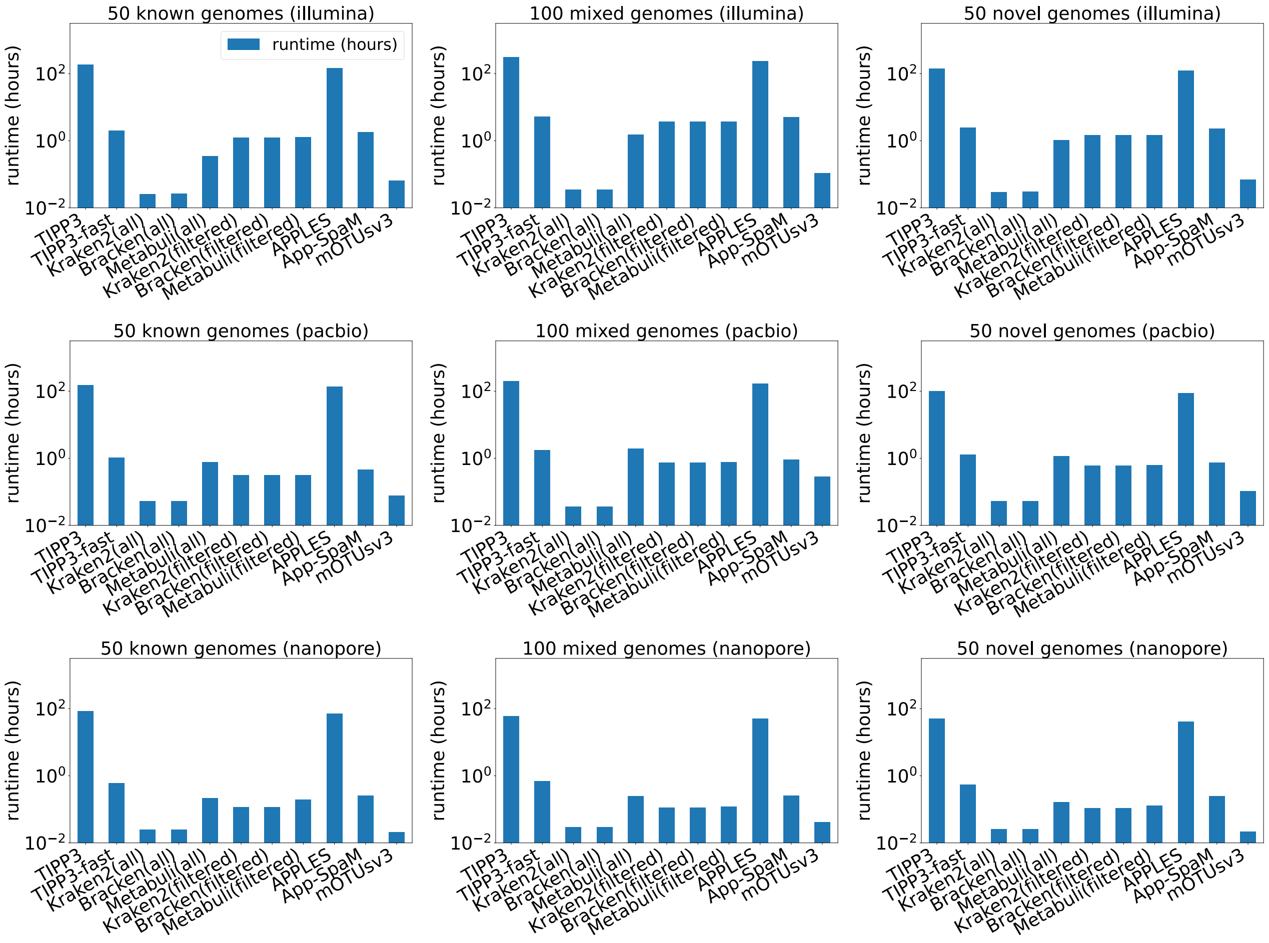

Fig U. Experiment 3: Runtime in hours of TIPP3, TIPP3-fast, Kraken2, Bracken, Metabuli, mOTUsv3, APPLES, and App-SpaM for Illumina, PacBio, and Nanopore simulated reads from three mock microbial communities with 50 known, 100 mixed, and 50 novel genomes.

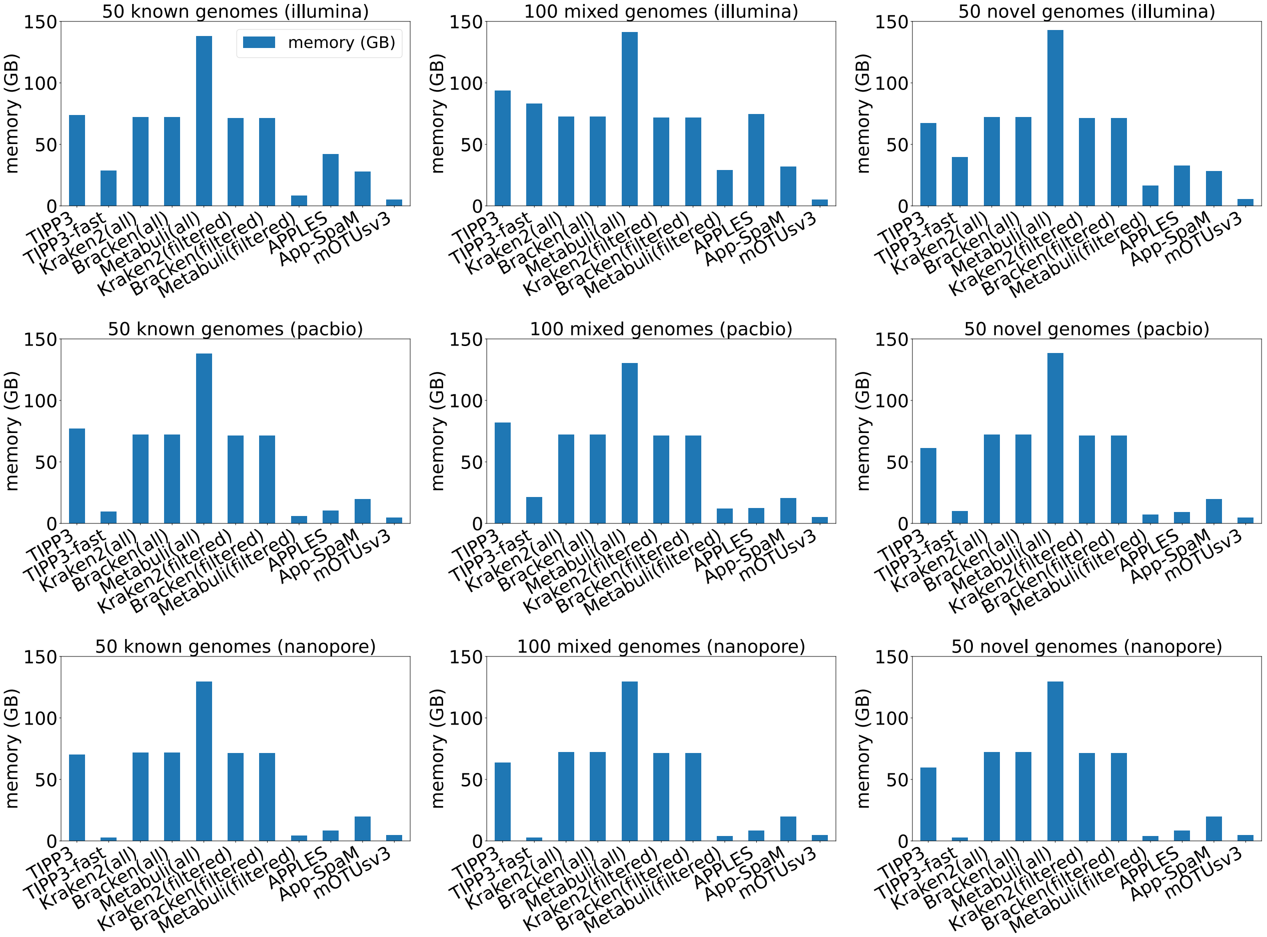

Fig V. Experiment 3: Maximum memory usage in GBs of TIPP3, TIPP3-fast, Kraken2, Bracken, Metabuli, mOTUsv3, APPLES, and App-SpaM for Illumina, PacBio, and Nanopore simulated reads from three mock microbial communities with 50 known, 100 mixed, and 50 novel genomes.

Table A. Experiment 3: Runtime in hours for all methods as shown in Fig U, excluding APPLES and AppSpaM.

community species	read type	TIPP3 (hours)	TIPP3-fast	Kraken2 (all)	Bracken (all)	Metabuli (all)	Kraken2 (filtered)	Bracken (filtered)	Metabuli (filtered)	mOTUsv3
50 known	Illumina	188.37	2.01	0.03	0.03	0.35	1.25	1.25	1.27	0.07
	PacBio	150.02	1.03	0.05	0.05	0.77	0.31	0.31	0.32	0.08
	Nanopore	83.06	0.61	0.02	0.02	0.21	0.12	0.12	0.19	0.02
100 mixed	Illumina	312.11	5.20	0.04	0.04	1.52	3.71	3.71	3.74	0.11
	PacBio	200.04	1.73	0.04	0.04	1.93	0.74	0.74	0.78	0.28
	Nanopore	59.97	0.69	0.03	0.03	0.24	0.11	0.11	0.12	0.04
50 novel	Illumina	143.02	2.44	0.03	0.03	1.05	1.48	1.48	1.48	0.07
	PacBio	101.27	1.26	0.05	0.05	1.14	0.60	0.60	0.62	0.10
	Nanopore	50.04	0.54	0.03	0.03	0.16	0.11	0.11	0.13	0.02

Table B. TIPP3 and TIPP3-fast runtime comparison in hours and the corresponding speedup by TIPP3-fast to TIPP3; average results are shown in the last row.

	TIPP3 (hours)	TIPP3-fast (hours)	speed up
50 known genomes (Illumina)	188.4	2.0	93.7
50 known genomes (PacBio)	150.0	1.0	146.1
50 known genomes (Nanopore)	83.1	0.6	137.2
100 mixed genomes (Illumina)	312.1	5.2	60.0
100 mixed genomes (PacBio)	200.0	1.7	115.8
100 mixed genomes (Nanopore)	60.0	0.7	86.7
50 novel genomes (Illumina)	143.0	2.4	58.6
50 novel genomes (PacBio)	101.3	1.3	80.4
50 novel genomes (Nanopore)	50.0	0.5	92.0
Average	143.1	1.7	93.7

G.4 Improving TIPP3 runtime performance

A key observation on TIPP3 and other tested methods is that TIPP3 is among the slowest and uses a high amount of memory. To improve the runtime and memory usage of TIPP3, we explored different modifications to the TIPP3 pipeline by changing one or more steps. Each TIPP3 variant is denoted as "<align method>+<placement method>" (using 38 marker genes) or "<align method>+<placement method>+<Xmarker>" (using X marker genes). For example, default TIPP3 uses WITCH for query alignment and pplacer-taxtastic for query placement and is denoted as witch+pplacer. TIPP3-fast uses BLAST for query alignment and BSCAMPP for query placement and is denoted as blast+bscampp. We compare these variants of TIPP3 on their profiling accuracy, runtime, and memory usage on Illumina and PacBio reads of the testing datasets (50 known, 100 mixed, and 50 novel genomes).

We showed the best variants in terms of profiling accuracy in Figure W and the corresponding runtime/memory usage in Figure X.

For reads from known genomes, all TIPP variants that use pplacer-taxtastic for read placement have similar profiling accuracy for Illumina or PacBio reads. Switching to BSCAMPP or SCAMPP for read placement leads to higher errors at all taxonomic levels observed on known reads. For the read alignment method, using WITCH results in slightly better accuracy than using BLAST, and the difference is more noticeable at lower taxonomic levels. One exception is at the species level for PacBio reads, where using BLAST is more accurate than using WITCH. For marker gene selection, using 20 instead of 38 marker genes does not impact profiling accuracy noticeably. 20 marker genes lead to slightly higher accuracy at the species level for both Illumina and PacBio reads, but we also observe slightly lower accuracy for family level and above of PacBio reads.

For reads from a mixture of known and novel genomes or entirely novel genomes, all TIPP3 variants have lower accuracy compared to known genomes. The relative performance resembled most observations on known genomes with a few differences. Switching to BSCAMPP or SCAMPP for read placement had similar accuracy to the other TIPP3 variants. Using 20 instead of 38 marker genes harmed profiling accuracy and was particularly noticeable on higher taxonomic levels (e.g., phylum and superkingdom).

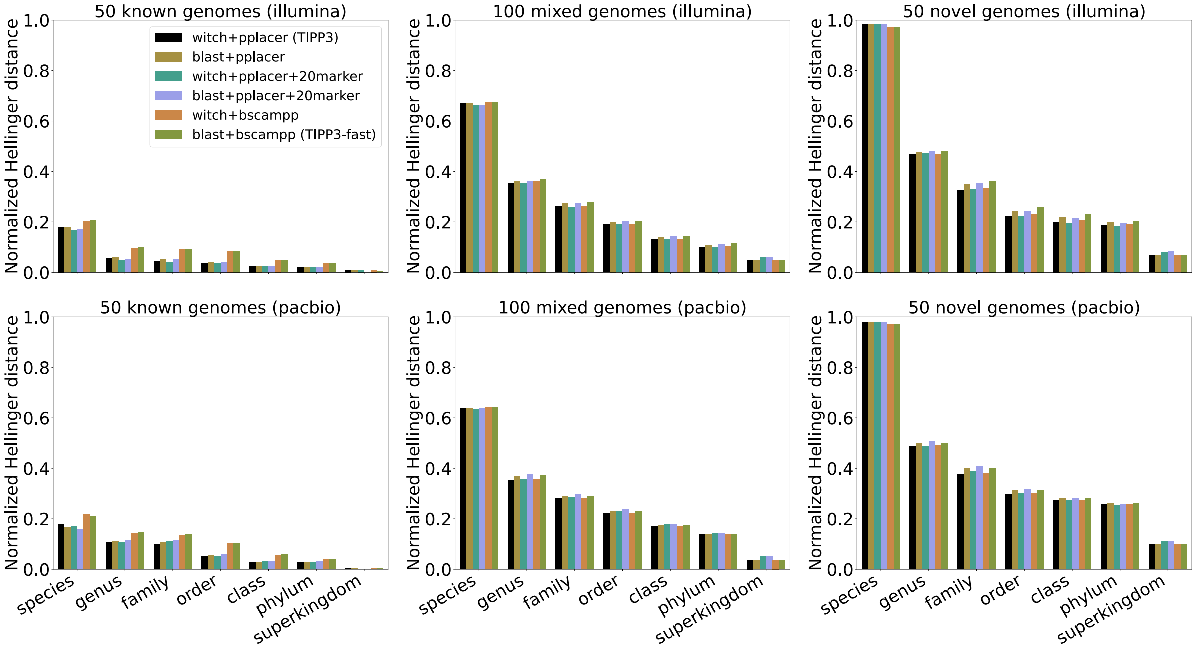

Fig W. Abundance profile accuracy by normalized Hellinger distance of TIPP3 variants for Illumina and PacBio simulated reads from known, mixed, and novel genomes. Each TIPP3 variant is denoted as "`<align method>`+`<placement method>`" (using 38 marker genes) or "`<align method>`+`<placement method>`+`<Xmarker>`" (using X marker genes). For example, TIPP3 uses WITCH for query alignment and pplacer-taxtastic for query placement and is denoted as "`witch+pplacer`".

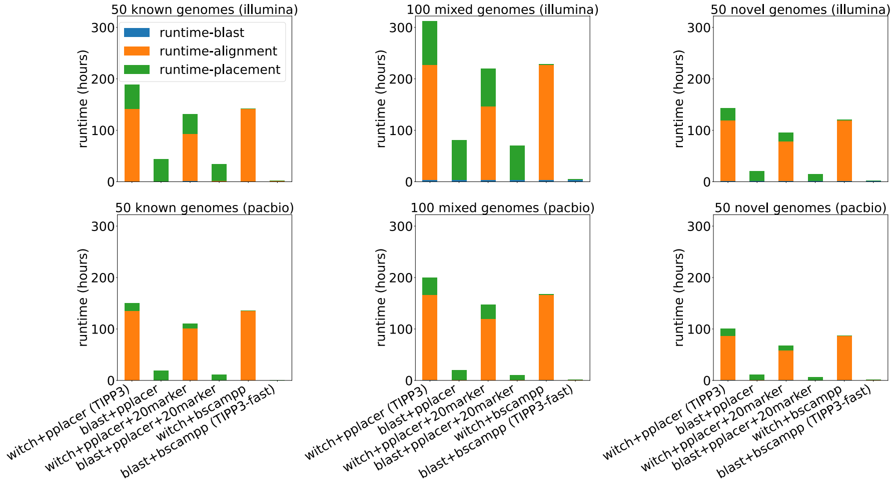

(a) Runtime (log-scale, hours).

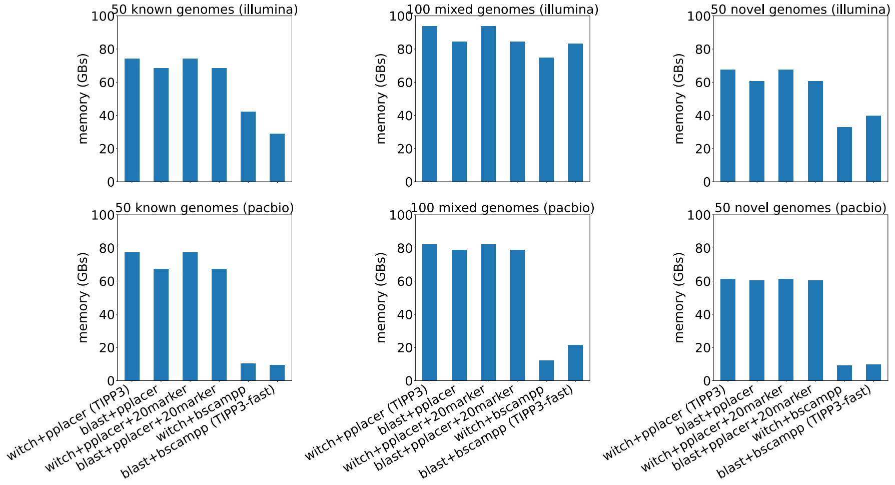

(b) Maximum memory usage (GBs).

Fig X. (a) Runtime in hours and (b) peak memory usage in GBs (bottom) of TIPP3 variants for Illumina and PacBio simulated reads from known, mixed, and novel genomes.

References

1. Shah N, Molloy EK, Pop M, Warnow T. TIPP2: metagenomic taxonomic profiling using phylogenetic markers. Bioinformatics. 2021;37(13):1839–1845. doi:10.1093/bioinformatics/btab023.

2. Smirnov V, Warnow T. MAGUS: Multiple sequence Alignment using Graph clUStering. Bioinformatics. 2021;37(12):1666–1672. doi:10.1093/bioinformatics/btaa992.

3. Stamatakis A. RAxML version 8: a tool for phylogenetic analysis and post-analysis of large phylogenies. Bioinformatics. 2014;30(9):1312–1313. doi:10.1093/bioinformatics/btu033.

4. Matsen FA, Kodner RB, Armbrust EV. pplacer: linear time maximum-likelihood and Bayesian phylogenetic placement of sequences onto a fixed reference tree. BMC Bioinformatics. 2010;11(1):538. doi:10.1186/1471-2105-11-538.

5. Hoffman N, Rosenthal C, Matsen E. taxtastic - python package; 2024. Available from: `https://github.com/fhcrc/taxtastic`.

6. Price MN, Dehal PS, Arkin AP. FastTree 2 – Approximately Maximum-Likelihood Trees for Large Alignments. PLOS ONE. 2010;5(3):e9490. doi:10.1371/journal.pone.0009490.

7. Kozlov AM, Darriba D, Flouri T, Morel B, Stamatakis A. RAxML-NG: a fast, scalable and user-friendly tool for maximum likelihood phylogenetic inference. Bioinformatics. 2019;35(21):4453–4455. doi:10.1093/bioinformatics/btz305.

8. Huang W, Li L, Myers JR, Marth GT. ART: a next-generation sequencing read simulator. Bioinformatics. 2012;28(4):593–594. doi:10.1093/bioinformatics/btr708.

9. Ono Y, Asai K, Hamada M. PBSIM: PacBio reads simulator—toward accurate genome assembly. Bioinformatics. 2013;29(1):119–121. doi:10.1093/bioinformatics/bts649.

10. Yang C, Chu J, Warren RL, Birol I. NanoSim: nanopore sequence read simulator based on statistical characterization. GigaScience. 2017;6(4):gix010. doi:10.1093/gigascience/gix010.

11. Kiełbasa SM, Wan R, Sato K, Horton P, Frith MC. Adaptive seeds tame genomic sequence comparison. Genome Res. 2011;21(3):487–493. doi:10.1101/gr.113985.110.

12. Wedell E, Shen C, Warnow T. BATCH-SCAMPP: Scaling phylogenetic placement methods to place many sequences; 2023. Available from: `https://www.biorxiv.org/content/10.1101/2022.10.26.513936v3`.

13. Wedell E, Cai Y, Warnow T. SCAMPP: Scaling Alignment-based Phylogenetic Placement to Large Trees. IEEE/ACM Transactions on Computational Biology and Bioinformatics. 2022; p. 1417–1430. doi:10.1109/TCBB.2022.3170386.

14. Chu G, Warnow T. SCAMPP+FastTree: Improving Scalability for Likelihood-based Phylogenetic Placement. Bioinformatics Advances. 2023; p. vbad008. doi:10.1093/bioadv/vbad008.

15. Barbera P, Kozlov AM, Czech L, Morel B, Darriba D, Flouri T, et al. EPA-ng: Massively Parallel Evolutionary Placement of Genetic Sequences. Systematic Biology. 2019;68(2):365–369. doi:10.1093/sysbio/syy054.
